# Blowing epithelial cell bubbles with GumB: ShlA-family pore-forming toxins induce blebbing and rapid cellular death in corneal epithelial cells

**DOI:** 10.1371/journal.ppat.1007825

**Published:** 2019-06-20

**Authors:** Kimberly M. Brothers, Jake D. Callaghan, Nicholas A. Stella, Julianna M. Bachinsky, Mohammed AlHigaylan, Kara L. Lehner, Jonathan M. Franks, Kira L. Lathrop, Elliot Collins, Deanna M. Schmitt, Joseph Horzempa, Robert M. Q. Shanks

**Affiliations:** 1 Department of Ophthalmology, University of Pittsburgh, Pittsburgh, PA United States of America; 2 Charles T. Campbell Laboratory of Ophthalmic Microbiology; 3 Center for Biological Imaging, University of Pittsburgh, Pittsburgh, PA United States of America; 4 Department of Natural Sciences and Mathematics, West Liberty University, West Liberty, WV United States of America; INSERM U1220, FRANCE

## Abstract

Medical devices, such as contact lenses, bring bacteria in direct contact with human cells. Consequences of these host-pathogen interactions include the alteration of mammalian cell surface architecture and induction of cellular death that renders tissues more susceptible to infection. Gram-negative bacteria known to induce cellular blebbing by mammalian cells, *Pseudomonas* and *Vibrio* species, do so through a type III secretion system-dependent mechanism. This study demonstrates that a subset of bacteria from the Enterobacteriaceae bacterial family induce cellular death and membrane blebs in a variety of cell types via a type V secretion-system dependent mechanism. Here, we report that ShlA-family cytolysins from *Proteus mirabilis* and *Serratia marcescens* were required to induce membrane blebbling and cell death. Blebbing and cellular death were blocked by an antioxidant and RIP-1 and MLKL inhibitors, implicating necroptosis in the observed phenotypes. Additional genetic studies determined that an IgaA family stress-response protein, GumB, was necessary to induce blebs. Data supported a model where GumB and *shlBA* are in a regulatory circuit through the Rcs stress response phosphorelay system required for bleb formation and pathogenesis in an invertebrate model of infection and proliferation in a phagocytic cell line. This study introduces GumB as a regulator of *S*. *marcescens* host-pathogen interactions and demonstrates a common type V secretion system-dependent mechanism by which bacteria elicit surface morphological changes on mammalian cells. This type V secretion-system mechanism likely contributes bacterial damage to the corneal epithelial layer, and enables access to deeper parts of the tissue that are more susceptible to infection.

## Author summary

Bacteria must overcome host defenses to cause infection. This is especially true for corneal infections where bacteria must penetrate the epithelium in order to gain access to the stroma where bacteria can rapidly multiply, induce inflammation, and cause vision loss. Members of the Enterobacteriaceae commonly cause contact lens associated infections, but the mechanisms by which they damage corneal cells are largely unknown. Here we present evidence that *Serratia marcescens* and *Proteus mirabilis* are able to induce dramatic morphological changes in mammalian corneal cells that correlates with rapid cellular death. Secretion of ShlA-like cytolysins via type V secretion was responsible for this phenotype, and this effect was regulated by the conserved Rcs phosphorelay stress response system, including IgaA-family protein GumB. This study provides a model for stress-mediated regulation of cytolysins that induce epithelial damage and promote ocular infection.

## Introduction

Some bacteria induce changes in human cell architecture through expression of virulence factors, which aid in bacterial internalization into cells and provide a more favorable niche for microbial colonization. Cell alterations include the formation of pedestals on intestinal cells by enteropathogenic and enterohaemorrhagic *Escherichia coli* strains through expression of type III secretion system (T3SS)-associated effector proteins that alter the actin cytoskeleton [1, 2]. Other bacteria create membrane ruffles, alterations that facilitate bacterial invasion into the mammalian cell [3]. Another type of mammalian cell surface alteration, known as bleb formation, appears following cellular injury [4, 5]. The bacterium *Pseudomonas aeruginosa* can induce bleb formation in airway and ocular cells. These blebs are similar to, but are more stable than, necrotic blebs and require a T3SS encoded by *P*. *aeruginosa*, and the ExoS and ExoY T3SS effector proteins [6, 7]. T3SS-dependent induction of membrane blebs on human cells was also reported for *Vibrio parahemolyticus* [8]. Large necrotic membrane blebs can also be induced by hydrogen peroxide and are thought to be a last-ditch effort by the cells to evade lysis. In this setting, they may serve as a storage unit to sequester damaged cellular components away from the cell or are simply a loss of cell homeostasis [5, 9, 10]. Blebs induced by membrane breach are hypothesized to be a result of the influx of calcium that activates hydrolytic enzymes capable of damaging the cellular cortex [11].

Gram-negative bacteria of the Enterobacteriaceae family, such as *Proteus mirabilis* and *Serratia marcescens*, cause nosocomial infections in neonates and immune compromised patients and contact lens associated complications in healthy individuals, including keratitis [12–17]. Microbial keratitis, or infection of the cornea, is a potentially blinding infection with a poor visual outcome, even when effective antibiotics are used to treat the infecting bacterium [14, 18]. Bacteria must overcome the epithelial cell layer in order to cause keratitis, and the killing of ocular surface cells is one mechanism bacteria could use to access the stromal layer that resides under the epithelium [19]. Therefore, we set out to study mechanisms by which keratitis causing bacteria damage the epithelium, which are largely unknown for the Enterobacteriaceae family of bacteria.

In this study, we observed that clinical keratitis isolates of *S*. *marcescens* cause bleb formation and cellular death in human ocular cells. However, *S*. *marcescens* bacterial genomes rarely encode genes for a T3SS, with strain FS14 [20], isolated from a leaf-cutter ant fungus garden, being the only strain described in the literature with a T3SS. These results suggested that *S*. *marcescens* has another mechanism to elicit these structural changes from the human epithelial cell, and we therefore employed a genetic screen to identify bacterial genes required for eliciting bleb formation. The role of the identified genes in bleb formation, cytotoxicity, and virulence was characterized using strains with deletion mutations and their corresponding complements, and a potential regulatory pathway was determined. We demonstrated that the Rcs stress response system controls expression of pore forming toxins secreted by a type V secretion system (T5SS) mechanism. The role of the T5SS-dependent cytolysin in bleb formation was validated using keratitis isolates of *P*. *mirabilis*, which suggests a novel conserved mechanism by which bacteria can induce cellular blebs to facilitate pathogenesis at epithelial surfaces.

## Results

### *S*. *marcescens* induces toxic membrane bleb formation by human corneal cells *in vitro*

**Fig 1:**
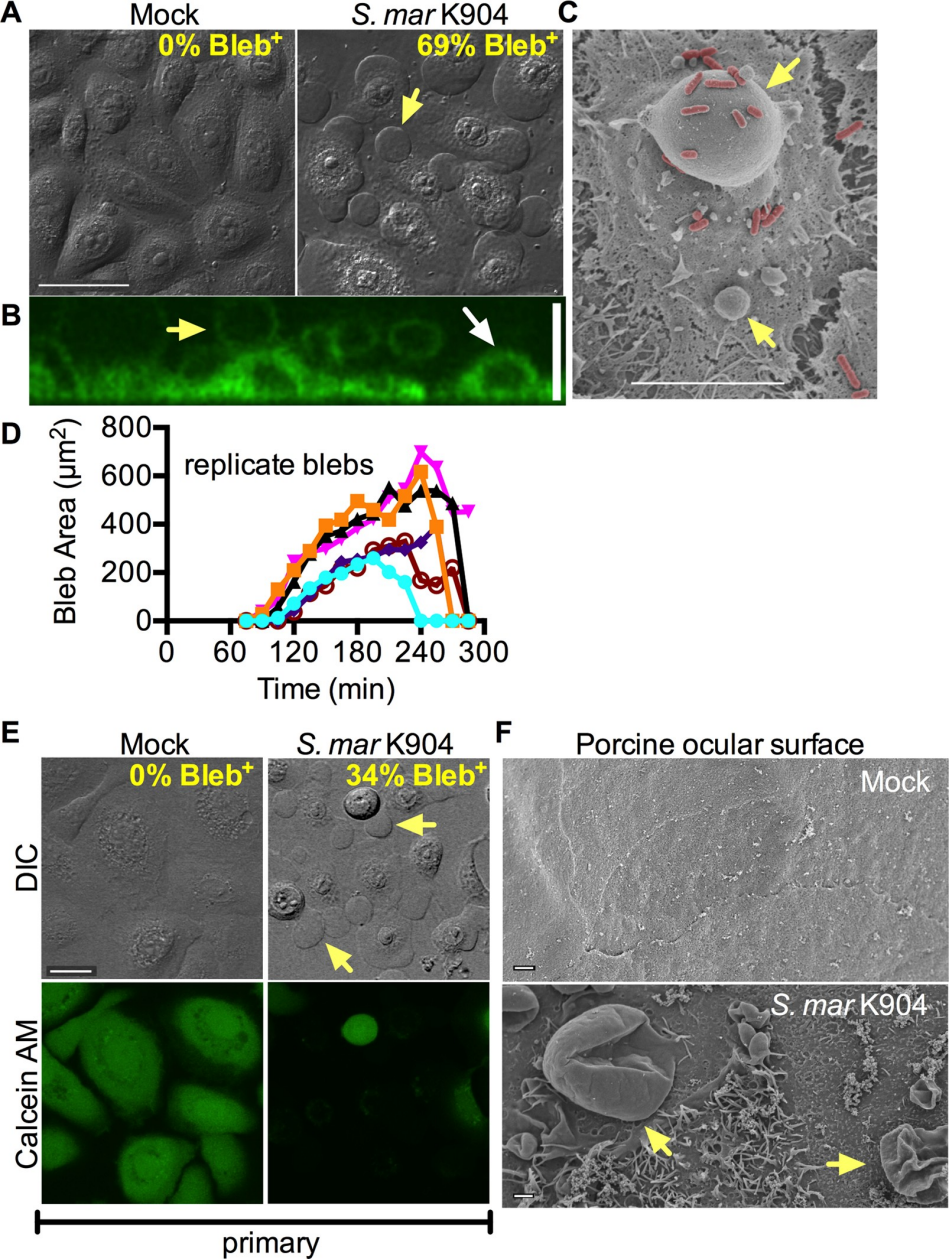
*S*. *marcescens* induces blebs and toxicity in human primary corneal cells and on porcine corneal tissue. (A) Confocal differential interference contrast (DIC) micrographs of human corneal cell line (HCLE) challenged with LB medium (mock) or with *S*. *marcescens* strain K904 (MOI = 200 for 2 h). Yellow arrows indicate a bleb extending from one of the cells. Size bar indicates 50 μm. (B) Confocal micrograph of HCLE cells challenged with *S*. *marcescens* strain K904 for 2 h and stained with a fluorescent membrane dye. The image shows a side projection of a z-stack of images. White arrow indicates a surface attached HCLE cell and the yellow arrow indicates a membrane bleb. Size bar is 20 μm. (C) SEM micrograph of blebs (yellow arrows) on a HCLE cell speckled with pseudocolored *S*. *marcescens* K904 bacteria (red). Size bar is 10 μm. (D) 2-D area of arbitrarily chosen blebs from 6 independent experiments tracked with video microscopy. (E) Primary corneal cells imaged by confocal microscopy with DIC and fluorescent imaging of the same cells stained with viability dye Calcein AM. Yellow arrows indicate blebs. Bar = 20 μm. Mock n = 48, K904 n = 22. (F) SEM micrograph of porcine corneas that had been exposed to naïve contact lenses (Mock, top) or contact lenses coated with wild-type *S*. *marcescens* (K904, bottom) prior to fixation. Blebs are indicated with yellow arrows.

Dramatic bleb formation on the cellular surface of a human corneal epithelial cell line (HCLE) was observed by microscopic analysis when *S*. *marcescens* contact lens-associated keratitis isolate K904 was co-incubated with the human cells (Fig 1A). Bleb formation was absent in HCLE cells exposed to bacterial growth medium (Mock) without bacteria (0%, n = 969 cells), whereas 69.5 ± 15.0% of HCLE cells challenged with *S*. *marcescens* K904 bacteria for 2 h (MOI = 200) produced blebs (n = 920 cells) (p<0.001, Fisher Exact test). Bleb formation frequency remained high when the MOI was 50 (95.4 ± 8.2%, n = 571), but reduced with an MOI = 10, (4.6%, n = 22). Confocal laser scanning microscopy and CellMask fluorescent membrane stain support that the bleb structures are extensions of HCLE plasma membranes and can become almost as large as the cells (Fig 1B). Scanning electron microscopy revealed *S*. *marcescens* bacteria associated with the membrane blebs (Fig 1C).

Live microscopic imaging of HCLE cells exposed to *S*. *marcescens* K904 produced membrane blebs starting before 120 minutes of co-incubation (compare S Movie 1 for Mock and S Movie 2 for *S*. *marcescens* K904, Fig 1D). Blebs were observed to grow over time, and then retract into the cell body as the cells rounded up (Fig 1D).

We tested whether the observed phenomenon was an artifact of using a specific human cell type, bacterial strain, or bacterial species. Testing bacterial strain specificity, we observed that 34 out of 34 *S*. *marcescens* strains derived from a variety of sources including environmental and clinical isolates induced bleb formation; these include reference strain Db11 and laboratory strain PIC3611 (S1A Fig). A variety of other species were tested for the ability to induce blebs in HCLE cells (MOI = ~200 for 2 h exposure). We found that *Proteus mirabilis* and *Edwardsiella tarda* were able to induce blebs during the 2 h time frame (S1B Fig), but no blebs were induced by tested strains of *Acinetobacter baumannii*, *Citrobacter frundii*, coagulase negative staphylococci, *Enterobacter aerogenes*, *Enterococcus faecalis*, *Klebsiella pneumoniae*, *Morganella morganii*, *Staphylococcus aureus* MRSA and MSSA, *Staphylococcus epidermidis*, *Streptococcus pneumoniae* [21], and *Stenotrophomonas maltophilia*. With *Pseudomonas aeruginosa*, keratitis isolate K900 [22] did induce blebs (S1B Fig) but wound isolate PAO1 [23] did not under the tested conditions.

Beyond the HCLE cell line, human primary corneal cells produced blebs in response to *S*. *marcescens* strain K904 (Fig 1E). With an MOI of 50, 34% of primary cells had a bleb (n = 22), as compared to 0% without bacterial challenge (n = 48). Calcein AM staining for intact, metabolically active cells suggested that the blebbing cells are no longer viable (Fig 1E). Similarly, *S*. *marcescens* strain K904 induced membrane bleb formation in airway epithelial cell line A549 (S2A Fig). *S*. *marcescens* causes contact lens associated keratitis, so we tested whether this effect could be seen on corneal tissue exposed to *S*. *marcescens* inoculated contact lenses. Strain K904 was introduced onto contact lenses and exposed to pig corneas *ex vivo* for 3 h. SEM analysis revealed extensive surface changes and membrane bleb formation on the porcine ocular surface on the *S*. *marcescens* exposed corneas, but not on control corneas bearing contact lenses without bacteria (Fig 1F).

### Genetic screen for mutant strains deficient in bleb induction

**Fig 2:**
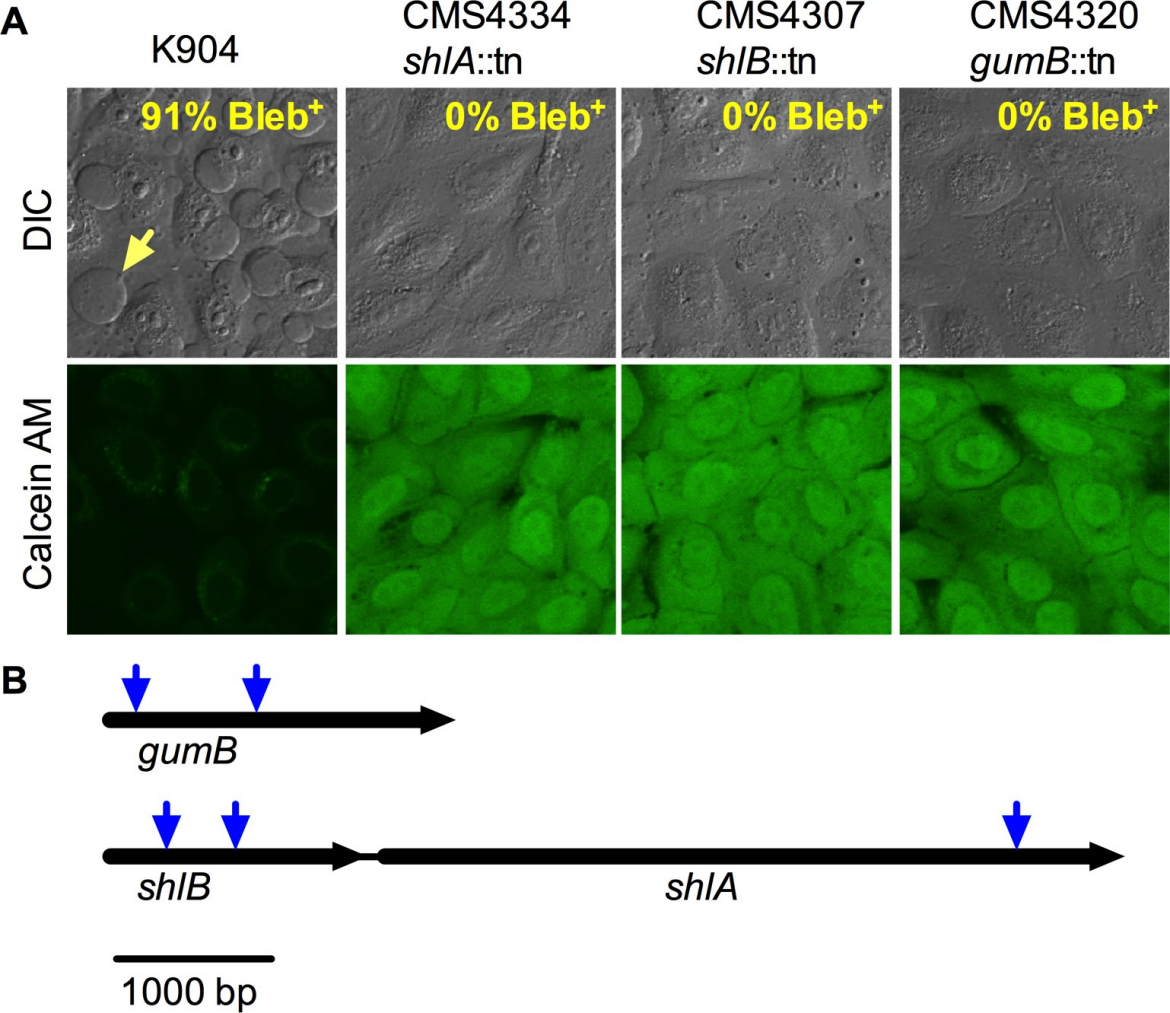
*S*. *marcescens* mutations that abrogate cytotoxicity in HCLE cells were isolated and mapped. (A) Confocal micrographs of primary human corneal epithelial cells challenged with *S*. *marcescens* strain K904 wild type and mutant strains isolated for being unable to induce bleb formation (MOI = 200 for 2 h). Three of five mutants are shown, with the other two having an indistinguishable effect on the corneal cells. Differential interference contrast (DIC) and calcein AM viability stained images are shown. (B) Genetic context of transposon insertion mutations that render *S*. *marcescens* unable to induce bleb formation in corneal epithelial cells. Downward facing blue arrows indicate transposon insertion sites. Size bar indicates 1000 base pairs.

The *S*. *marcescens* strain K904 genome was sequenced (Genbank PRJNA243053) with no evidence for a type III secretion system (T3SS). Because a T3SS-independent mechanism was therefore implicated, transposon mutagenesis was performed to elucidate the bacterial factors required for this phenotype. 6,920 mutants were screened for the inability to induce bleb formation in HCLE cells and kill the cells as judged by Calcein AM staining. Five mutants were reproducibly defective in bleb induction, which we confirmed with primary corneal epithelial cells. For K904 91.2±4.9% (n = 334) of cells had blebs, compared to 0% for the mutants (n≥160) (Fig 2A). The mutations were mapped to two loci. Three were in the *shlBA* operon, with two in *shlB* at base pair 378 and 825 out of the 1680 base pair gene, and one in *shlA* at base pair 4063 out of the 4824 base pair gene (Fig 2B). Two other mutations mapped to different locations in the *gumB* gene at base pairs 170 and 957 out of the 2136 base pair gene (Fig 2B). The *shlBA* operon codes for a type Vb secretion system with ShlB being an outer membrane transporter and ShlA its cognate surface-associated and secreted cytolysin [24]. The *gumB* gene is a recently described member of the IgaA family involved in bacterial stress response that confers pleiotropic phenotypes when mutated [25]. IgaA is an inner membrane protein that influences *Salmonella* virulence in rodent infection models [26] and controls the Rcs stress response transcriptional system.

### *shlA* and *shlB* genes are necessary, and ShlA is sufficient, for bleb induction

Fifteen of the 34 bleb-inducing *S*. *marcescens* clinical isolates were selected arbitrarily among isolates that had caused different types of ocular infections. These were subject to PCR analysis for the *shlA* gene. Strain PIC3611 was used as a positive control and an *shlBA* deletion variant of PIC3611 was used as a negative control. DNA samples from all tested strains, except the deletion mutant, produced an amplicon consistent with the *shlA* gene (S3 Fig). This result is consistent with *shlA* being a conserved gene in *S*. *marcescens*, and likely responsible for bleb induction by the tested strains.

**Fig 3:**
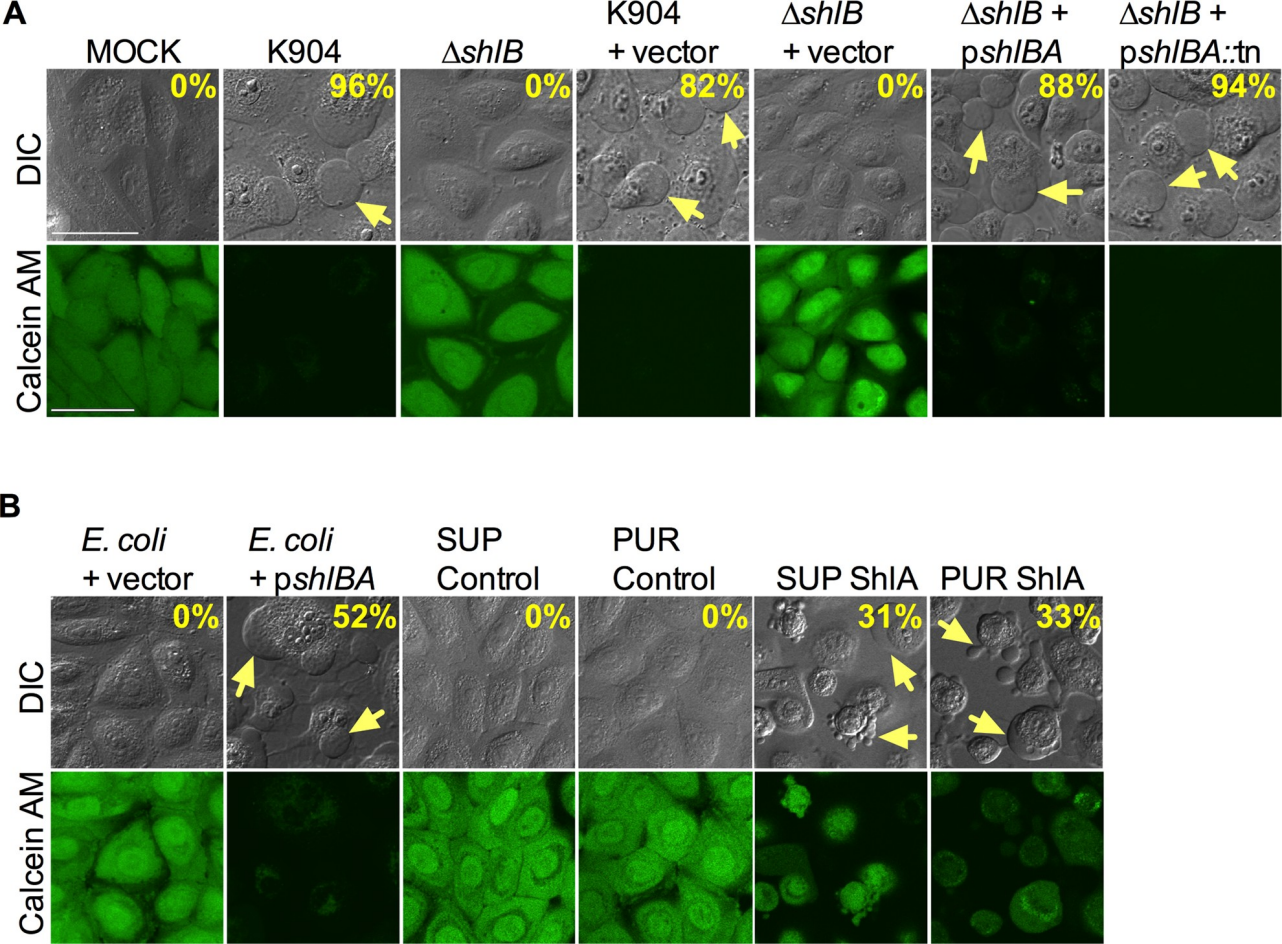
*S*. *marcescens shlBA* operon is necessary, and ShlA is sufficient, for bleb and cytotoxicity induction. Confocal micrographs of HCLE cells imaged with differential interference contrast (DIC) and calcein AM viability stain. Yellow arrows indicate blebs extending from corneal cells. The percent of bleb positive cells induced by the indicated treatment are shown. (A) Confocal micrographs of HCLE cells with *S*. *marcescens* strain K904 and mutant strains (MOI = 50, 2 h incubation). Vector = pMQ125 or pMQ131; p*shlBA* = pMQ541; p*shlBA*::tn = pMQ591. (B) Microscopic evaluation of HCLE cells exposed to *E*. *coli* (Top10) with a vector (pMQ175) or *shlBA* expressing plasmid (pMQ492) at MOI = 50 for 2 h. Cells were alternatively exposed for 3 h to sterile-filtered supernatants from *E*. *coli* with the pMQ175 (SUP Control) or pMQ492 (SUP ShlA) plasmids, or partially purified ShlA-containing supernatant fractions from *E*. *coli* with the vector negative control (PUR Control) or with pMQ492 (PUR ShlA). Vector = pMQ125; p*shlBA* = pMQ492.

To further test the necessity of this this operon in bleb induction by *S*. *marcescens*, a deletion allele of the *shlB* gene was constructed in *S*. *marcescens* strain K904. The Δ*shlB* mutant strain was completely defective in the ability to induce blebs and kill HCLE cells in our test conditions (Fig 3A). Addition of the *shlBA* operon expressed from the *nptII* promoter on a plasmid (p*shlBA*) complemented the Δ*shlB* mutant phenotype supporting that the defect was due to mutation of *shlB* and not an unknown mutation elsewhere on the chromosome or a polar effect on adjacent genes (Fig 3A). The pMQ591 plasmid (p*shlBA*::tn), which has the *shlBA* operon with a transposon insertion in *shlA* at base pair 4063, was able to restore bleb formation to the *S*. *marcescens* Δ*shlB* strain, providing genetic evidence that *the* Δ*shlB* mutation in strain CMS4236 was nonpolar, since the active ShlA protein must come from the chromosomal copy of *shlA* (Fig 3A). A resazurin fluorescence-based assay was used as a second method to validate the cytotoxic phenotypes demonstrated in this study using calcein AM staining (S4A and S4B Fig). We observed consistent results, including the lack of cytotoxicity caused by the Δ*shlB* mutant and the restoration of cytotoxicity using complementing plasmids (MOI 10 and 200) (S4A and S4B Fig). Thus, even though *shlA* may be expressed in the Δ*shlB* mutant, the ShlA protein is not secreted without the ShlB transporter, as was previously shown in other strains [24].

Importantly, *shlBA* expression was sufficient to confer bleb-induction ability to the *E*. *coli* laboratory strain S17-1 λ-*pir* that is normally unable to generate blebs (Fig 3B and S2 Fig). This result suggested that ShlA may be sufficient to induce bleb formation. Further evidence supporting that ShlA is sufficient for bleb induction came from observations that partially purified ShlA expressed from *E*. *coli* could induce bleb formation; 31±3% of HCLE cells exposed to filtered supernatants (n = 75) and 33±15% (n = 208) of cells challenged with purified ShlA exhibited blebs (Fig 3B). This is in sharp contrast to the absence of blebs in cells challenged with preparations made from *E*. *coli* harboring the control vector without *shlBA* (n≥80 cells, Fig 3B). *E*. *coli* expressing p*shlBA*::tn with the transposon mutation in *shlA*, noted above to contain a null *shlA* allele, was unable to induce blebs or kill HCLE cells (S2B Fig), which supports the conclusion that ShlA rather than ShlB is required for bleb induction. Combined, these data support a working model that the T5SS ShlB and cytolysin ShlA are the *S*. *marcescens* virulence factors responsible for induction of membrane blebs in mammalian cells and suggest that GumB is a regulator of *shlBA* expression.

### A ShlA-like, T5SS-dependent, cytolysin from *P*. *mirabilis* is necessary for bleb induction

**Fig 4:**
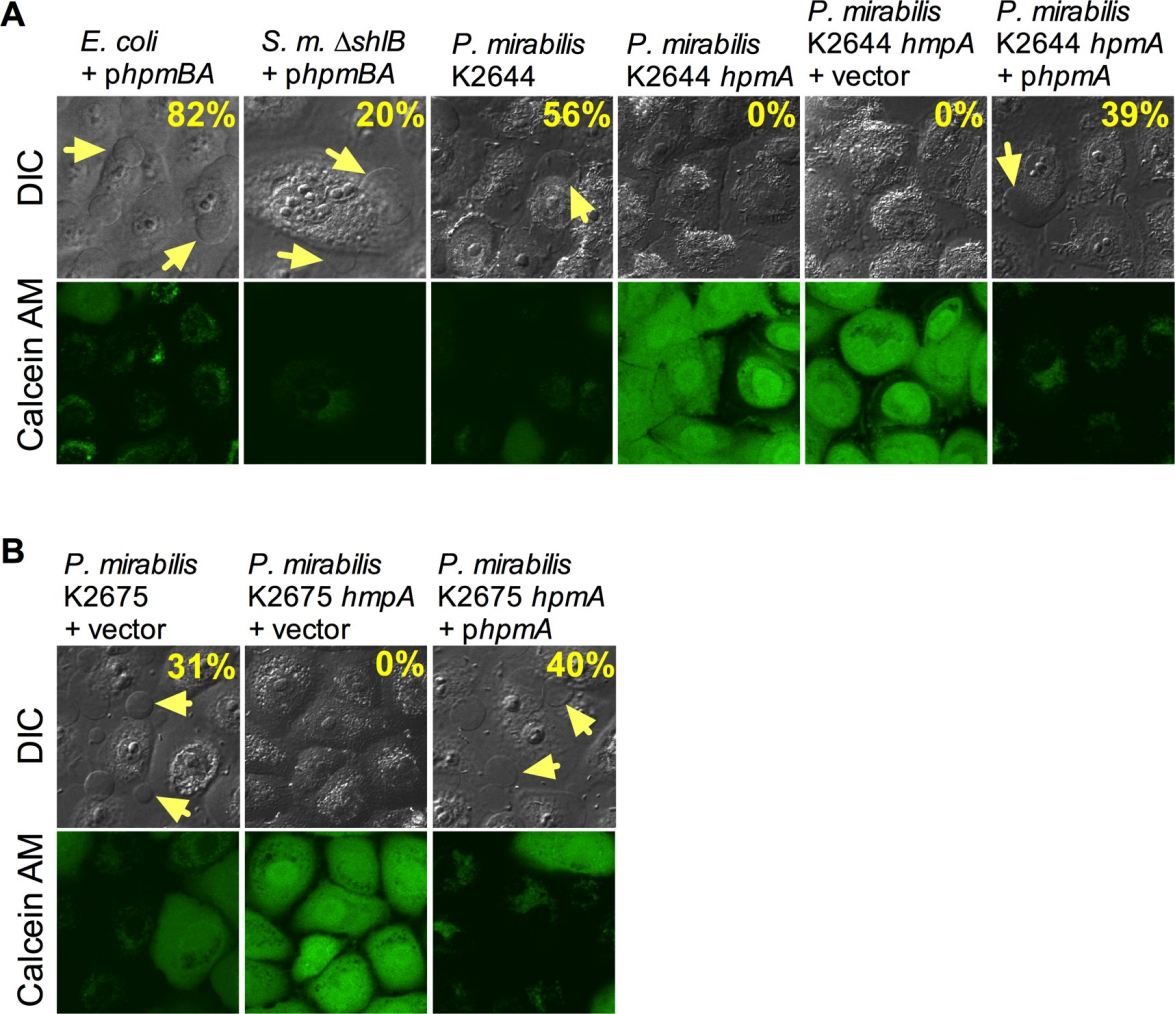
A *shlBA*-like Type Vb secretion system operon from *P*. *mirabilis* induces blebbing and cytotoxicity. Confocal micrographs of HCLE cells imaged with differential interference contrast (DIC) and calcein AM viability stain. Yellow arrows indicate blebs extending from corneal cells. (A) Confocal micrographs of HCLE cells with *E*. *coli* strain (Top10), *S*. *marcescens* (*S*. *m*.) Δ*shlB*, and *P*. *mirabilis* keratitis isolate K2644 and isogenic *hmpA* mutant strain (MOI = 50, 2 h incubation, 1 hour with *E*. *coli*). (B) As in (A), using *P*. *mirabilis* keratitis isolate K2675. p*hpmBA* = pMQ601; vector = pMQ132; p*hpmA* = pMQ602.

Since *P*. *mirabilis* is able to induce bleb formation in HCLE cells and its genome contains an *shlBA*-like virulence operon *hpmBA* [27], we tested whether this operon could induce bleb formation. HpmA is 44% identical to ShlA at the amino acid level and HpmB/A constitute a Type Vb secretion pair analogous to ShlBA. Induced expression of the *hpmBA* operon from a plasmid was able to confer the bleb-formation phenotype to *E*. *coli* (82±9% blebs, n = 156, Fig 4A). The *hpmBA* plasmid could complement the Δ*shlB* mutation in *S*. *marcescens*; there were fewer blebs than wild type treated cells (20±17%, n = 287), but the cells appeared to be dead (Fig 4A).

To further verify the importance of HpmA in bleb formation, the chromosomal *hpmA* gene of *P*. *mirabilis* was mutated in two clinical keratitis isolates K2644 and K2675, with 56±12% (n = 122) and 39±7% (n = 219) bleb formation, respectively, (Fig 4A). Unlike the wild-type parental strains, isogenic *hpmA* mutant strains were defective in bleb formation and toxicity, and these phenotypes could be complemented by expression of the *hpmA* gene from a plasmid. Zero percent bleb formation was observed from corneal cells treated with the *hpmA* mutants with the vector alone (n≥140). For cells exposed to the *hpmA* mutants with the *hmpA* plasmid, ~40% had blebs (39±12%, n = 208 for strain K2644 and 40±10%, n = 187 for strain K2675, Fig 4A and 4B). Together, these data indicate that T5SS secreted cytolysins of the ShlBA family represent a conserved mechanism by which bacteria elicit rapid cell death and dramatic morphological changes in human cells.

### GumB is required for bleb formation because of *shlBA* regulatory activity

**Fig 5:**
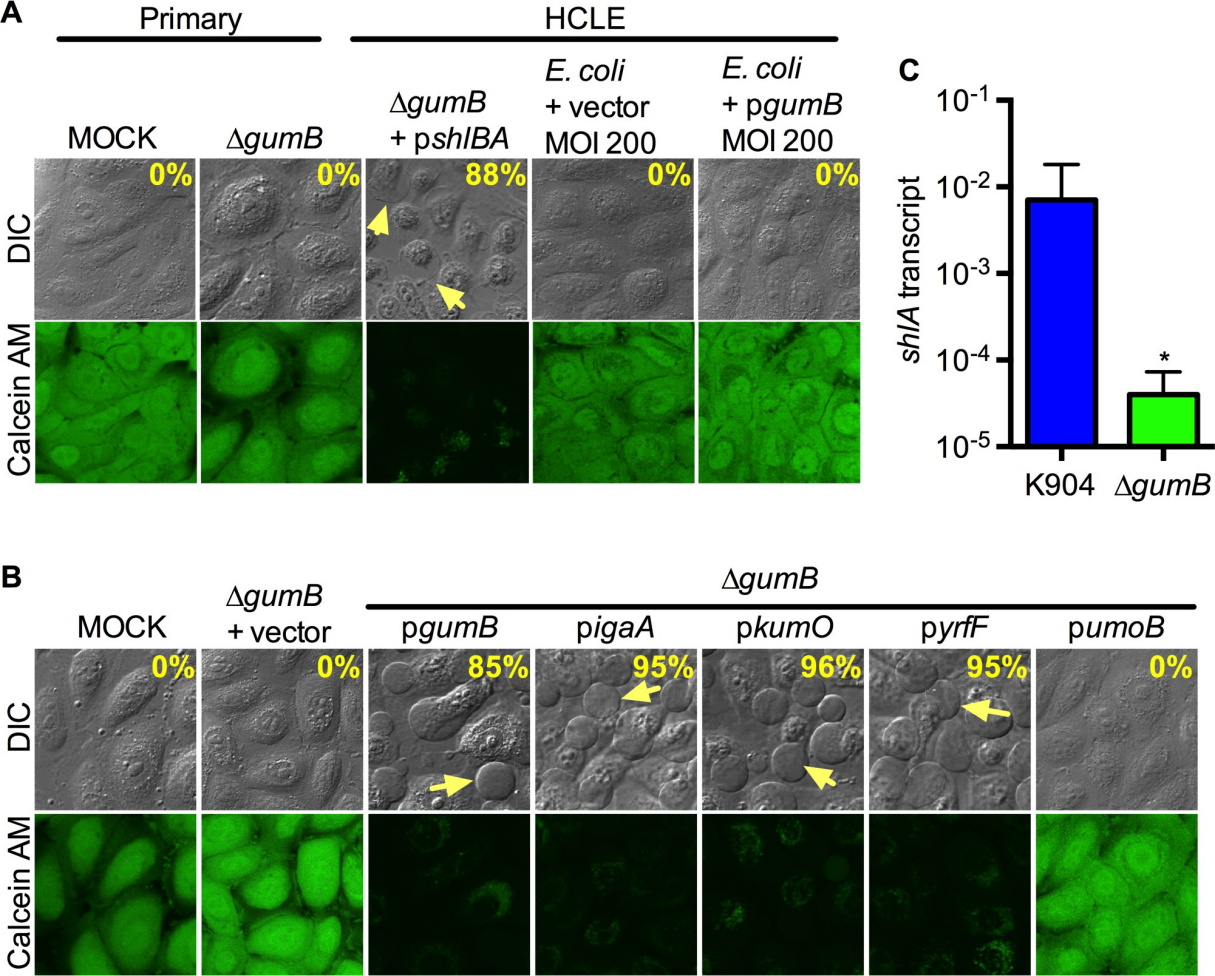
*S*. *marcescens gumB* gene is necessary but not sufficient for the bleb and cytotoxicity induction, and the *gumB* mutant strain defect is complemented by *igaA*-family genes and *shlBA*. (A,B) Confocal micrographs of primary human corneal and HCLE cells imaged with differential interference contrast (DIC) and calcein AM viability stain after exposure to bacteria for 2 h at MOI = 50, except where noted. Yellow arrows indicate blebs extending from corneal cells. (A) Confocal micrographs of HCLE cells with *S*. *marcescens* strain K904, mutant strains, or *E*. *coli* strain EC100D *pir-116*. Vector = pMQ132; p*shlBA* = pMQ541; p*gumB* = pMQ480. The percent of bleb positive cells induced by select bacteria are shown. (B) HCLE cells exposed to the *gumB* mutant strain (MOI = 50) with plasmid-borne *igaA*-family genes from *S*. *marcescens* (p*gumB* = pMQ480), *S*. *enterica* (p*igaA* = pMQ530), *K*. *pneumoniae* (p*kumO* = pMQ529), *E*. *coli* (p*yrfF* = pMQ531), or *P*. *mirabilis* (p*umoB* = pMQ600). Vector = pMQ132. (C) Relative gene expression using the ΔΔCT method depicts *shlA* transcript levels in the wild type (K904) and Δ*gumB* mutant strains at OD_600_ = 3; *p = 0.0286, Mann-Whitney test.

Similar to the *gumB* transposon mutant (Fig 2A), strain K904 with a deletion of the *gumB* gene was unable to induce blebs or kill primary corneal or HCLE cells (0% cells had blebs, n = 159 cells) (Fig 5A). The same trend was observed when the *gumB* gene was deleted from the *S*. *marcescens* reference and insect pathogen strain Db11 [28] (S1A Fig). Plasmid-based expression (*lac* promoter) of *gumB* (85±12%, n = 280) or IgaA-family genes from *Escherichia coli* (*yrfF*, 95±1%, n = 175), *Salmonella* Typhimurium (*igaA*, 95±5, n = 175), and *Klebsiella pneumoniae* (*kumO*, n = 96±1, n = 227) was able to restore bleb-induction ability to the Δ*gumB* mutant, which supports the notion that the function of IgaA-family proteins is highly conserved (Fig 5B). The *umoB* gene from *P*. *mirabilis* was unable to complement the Δ*gumB* mutant, suggesting that it differs enough structurally from GumB as to not replace protein-protein interactions necessary for GumB function in *S*. *marcescens* (Fig 5B and, 0%, n = 123). Additionally, expression of *gumB* in *E*. *coli* did not enable *E*. *coli* to induce blebs (Fig 5A) (0%, n = 82 for vector and 225 for p*gumB*), suggesting that GumB is necessary, but insufficient for the bleb-induction phenomenon.

We tested whether *shlA* expression was reduced in the *gumB* mutant using qRT-PCR, and observed a 100-fold reduction in transcript (Fig 5C). This finding suggests that *gumB* mutant strains likely are defective in the ability to induce blebs because they do not produce adequate ShlA cytolysin. To test this model, the constitutive *shlBA* expression plasmid was introduced into the *gumB* mutant. The resulting strain induced blebs and was highly cytotoxic (Fig 5A and S4 Fig), which indicates that artificial upregulation of *shlBA* bypasses the GumB-mediated regulation required for this virulence function.

### GumB regulates bleb formation and virulence through the Rcs system

**Fig 6:**
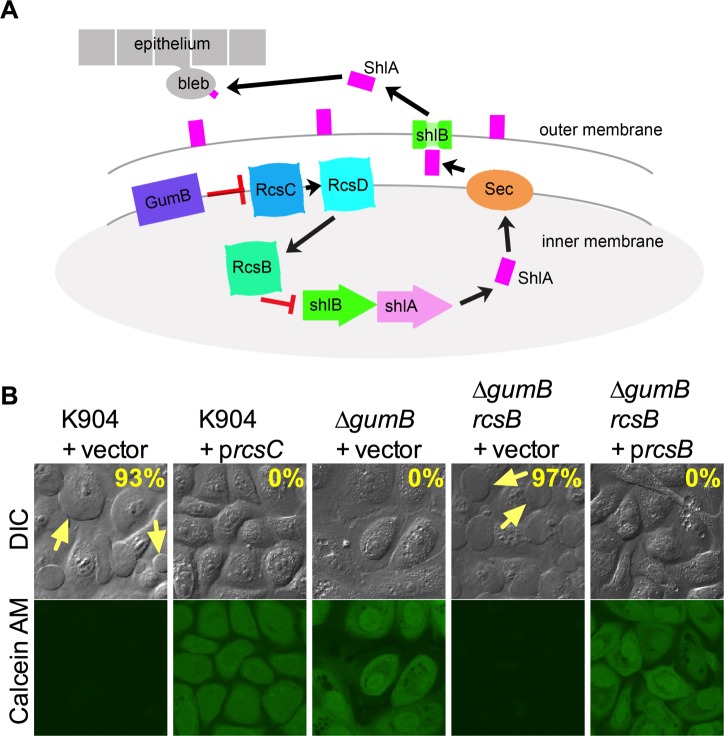
GumB regulation of bleb formation and cytotoxicity requires the Rcs signaling system. (A) Model for the regulatory circuit through which GumB functions to regulate epithelial cell bleb formation, based on this study and DiVenanzio, et al [58]. GumB inhibits (red stop bar) the Rcs-phosphorelay system through which the response regulator RcsB inhibits *shlBA* expression. The ShlA cytolysin is secreted through the outer membrane by ShlB and is maintained on the bacterial outer membrane or released into the environment where it can form pores in mammalian cell membranes and stimulate bleb formation and cellular death. (B) Confocal micrographs of HCLE cells imaged with differential interference contrast (DIC) and calcein AM viability stain after exposure to bacteria for 2 h at MOI = 50. Yellow arrows indicate blebs extending from corneal cells. Multicopy expression of the *rcsC* histidine sensor kinase gene confers *gumB*-like phenotypes to the wild type (p*rcsC* = pMQ514). The Δ*gumB* mutant bleb-phenotype is suppressed by mutation of the *rcsB* response regulator gene, and this effect can be complemented by the wild-type *rcsB* gene on a plasmid (p*rcsB* = pMQ614).

Reports indicate that IgaA-family proteins inhibit the Rcs phosphorelay system in other genera from the Enterobacteriaceae family [29, 30]. The Rcs system is a multicomponent version of a two-component transcription factor system involved in responses to extracellular and envelope stress [31, 32] (Fig 6A). The core Rcs system is composed of sensor histidine kinase RcsC, an intermediate phosphoprotein RcsD, and the RcsB response regulator [32]. Therefore, one would predict that if the Rcs system is derepressed in an IgaA-family protein mutant strain (Δ*gumB*), then elevated expression of Rcs system components in the wild-type strain could mimic the *gumB* mutant phenotypes (Fig 6A). To test this prediction, the *rcsC* gene was placed under control of the *E*. *coli lac* promoter on a medium-copy plasmid in the wild-type strain, K904. We observed *gumB* mutant strain-like phenotypes for the wild type with the *rcsC* multi-copy plasmid, such as reduction of pigmentation and mucoid colony morphology, which supports that multicopy expression of *rcsC* was activating the Rcs system akin to mutation of *gumB* that also prevents pigmentation (S5 Fig). HCLE cells exposed to strain K904 with the *rcsC* expression plasmid, but not the vector control, were defective in inducing bleb formation and cytotoxicity (Fig 6B). 93±6% bleb formation was observed in cells exposed to the K904 wild-type with vector control (n = 208), whereas those challenged with K904 with p*rcsC* produced no blebs (0%, n = 454).

The proposed model (Fig 6A) also suggests that inactivation of the Rcs system in a *gumB* mutant strain should restore toxicity, bleb induction ability, and pigment production. The gene for the RcsB response regulator was mutated in the Δ*gumB* strain background. In order to interrogate the model and validate the strains, Rcs regulation of pigmentation was analyzed. The *rcsB* mutation reversed the *gumB* mutant pigment defect (S5 Fig), which supports that RcsB acts downstream of GumB (S6 Fig). We also observed that the *gumB* mutant strain pigment defect could be restored through complementation with wild-type *rcsB* expression from a plasmid (S5 Fig), which further supports the validity of the mutation and plasmid. With regards to host-pathogen interactions, the Δ*gumB rcsB* double mutant strain was indistinguishable from the *S*. *marcescens* K904 parental strain for bleb induction and cytotoxicity to HCLE cells (Fig 6B). Importantly, the Δ*gumB* *rcsB* double mutant strain toxicity and bleb inducing phenotypes could be complemented with *rcsB* on a plasmid (Fig 6B). Zero blebbing cells were counted with the *gumB* mutant with the vector control (n = 141); 94±6% of cells had blebs in the *gumB rcsB* with vector group (n = 252), and 0% of cells had blebs when exposed to the *gumB rcsB* mutant complemented with p*rcsB* (n = 338). Together, these data indicate that bleb formation regulation by GumB requires a functional Rcs system, and that activation of the Rcs system prevents *S*. *marcescens* from inducing bleb formation and cytotoxicity to epithelial cells.

### *S*. *marcescens-*induced epithelial blebbing and toxicity is due to ShlA-mediated pore formation and resultant necroptosis

**Fig 7:**
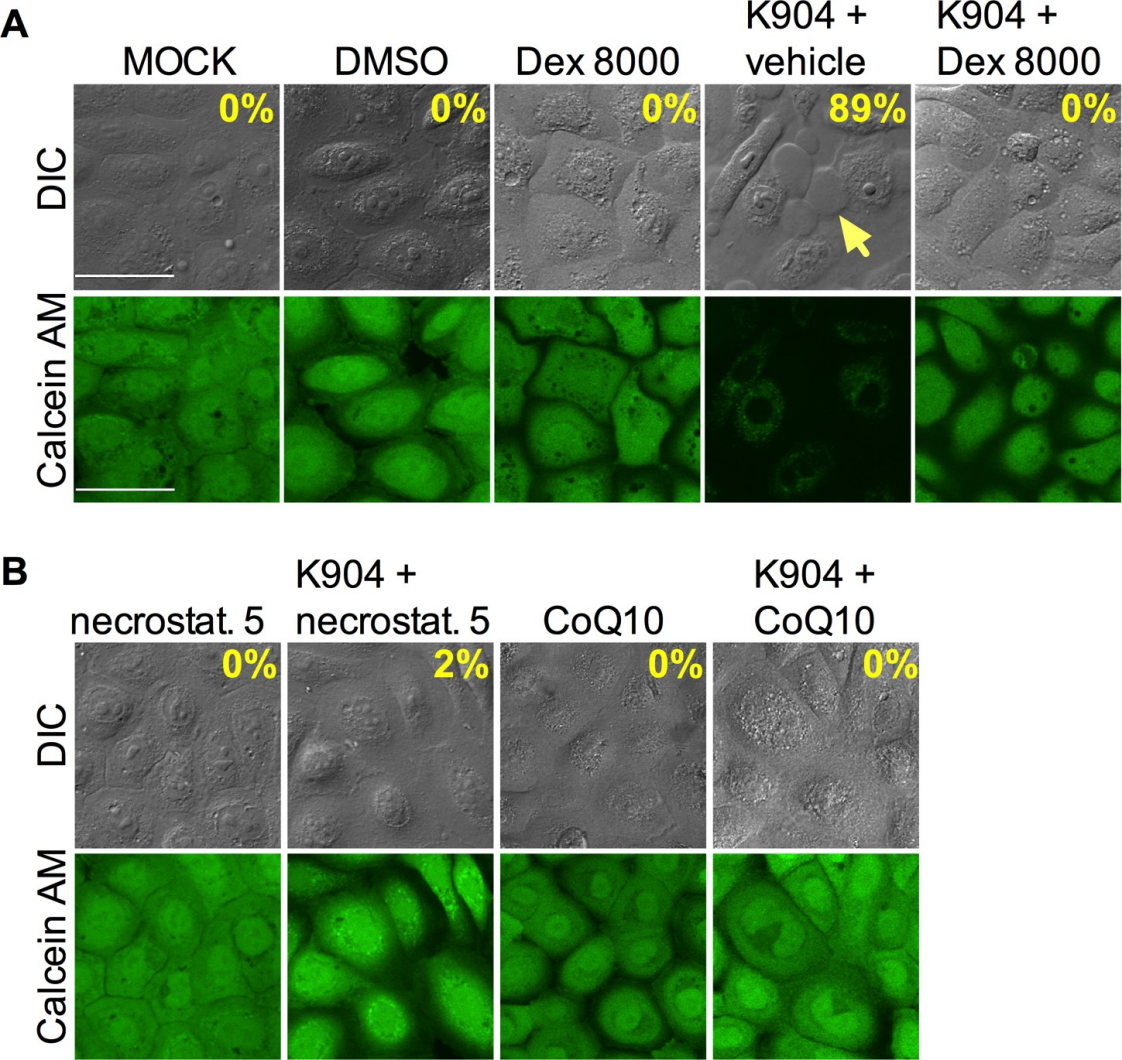
ShlA induces bleb formation and cytotoxicity via pore formation and subsequent necroptosis. Confocal micrographs HCLE cells imaged with differential interference contrast (DIC) and calcein AM viability stain after exposure to bacteria for 2 h at MOI = 50. Yellow arrows indicate blebs extending from corneal cells. HCLE cells were exposed to *S*. *marcescens* K904 (MOI = 50 for 2 h) with either (A) dextran sulfate 8000 (30 mM, occludes pores caused by pore-forming toxins), (B) necrostatin 5 (100 μM, an inhibitor of necroptosis), or coenzyme Q10 (CoQ10, 0.1 μM, an antioxidant,) or an equal volume of vehicle DMSO incubated with cells for 1 h prior to challenge.

Next, we investigated the mechanism of cellular death induced by ShlA-like cytolysins in corneal epithelium. Previous studies have shown that osmoprotectants can prevent bacterial T3SS-mediated bleb formation and necroptosis [33, 34]. The osmoprotectant sorbitol (300 mM) was able to reduce bleb formation from 89% (n = 225) for HCLE cells exposed to *S*. *marcescens* (strain K904) to 10% (n = 409) for those exposed to *S*. *marcescens* and sorbitol (p<0.001). Dextran, a branched polysaccharide, is able to prevent cellular lysis induced by purified streptolysin O and other pore forming toxins, including ShlA, by occluding pores introduced by such toxins [35–38]. Here, dextran was found to reduce cells with blebs from 94.5% (n = 383) with *S*. *marcescens* challenge MOI = 50 or 200 to 0 or 4.4% (n = 397/544) with *S*. *marcescens* and dextran, a significant reduction (p<0.0001, Fisher’s Exact) (Fig 7A). These data suggest that the membrane pore introduced by ShlA’s pore forming domain is responsible for the bleb and cytotoxicity phenotypes.

It has been demonstrated that intracellular *S*. *marcescens* can initiate the type of programmed cell death known as necroptosis in macrophages in a ShlA-dependent manner [39]. Oxidative stress plays a major role in necroptosis, so we tested whether the antioxidant coenzyme-Q10 (0.1 μM), had an impact on *S*. *marcescens* induced damage. CoQ10 prevented *S*. *marcescens*-induced cytotoxicity and bleb formation (0% blebs, n = 146) (Fig 7B), whereas DMSO alone did not alter the ability of *S*. *marcescens* to induce blebs (89±6%, n = 121) (Fig 7A).

We tested whether blocking of necroptosis using the RIP-1 inhibitor necrostatin 5 could alleviate *S*. *marcescens*-induced phenotypes; a strong reduction in bleb formation (2±3%, n = 316) and cytotoxicity was observed (Fig 7). Necrostatin 5 itself did not produce a bleb or cytotoxicity phenotype (0% blebs, n = 97), nor did the vehicle (DMSO), 0% blebs, n = 115). An inhibitor that targets the major regulator of necroptosis, the mixed lineage kinase domain-like protein (MLKL), was tested [39, 40]. The MLKL inhibitor GW806742X produced a dose-dependent reduction in bleb formation by HCLE cells challenged with wild-type *S*. *marcescens* (S7 Fig). Together, these data suggest that *S*. *marcescens*-induced necroptosis in response to ShlA-mediated pore formation is responsible for bleb formation and cellular death.

### *gumB* is required for virulence in a ShlA-dependent manner

**Fig 8:**
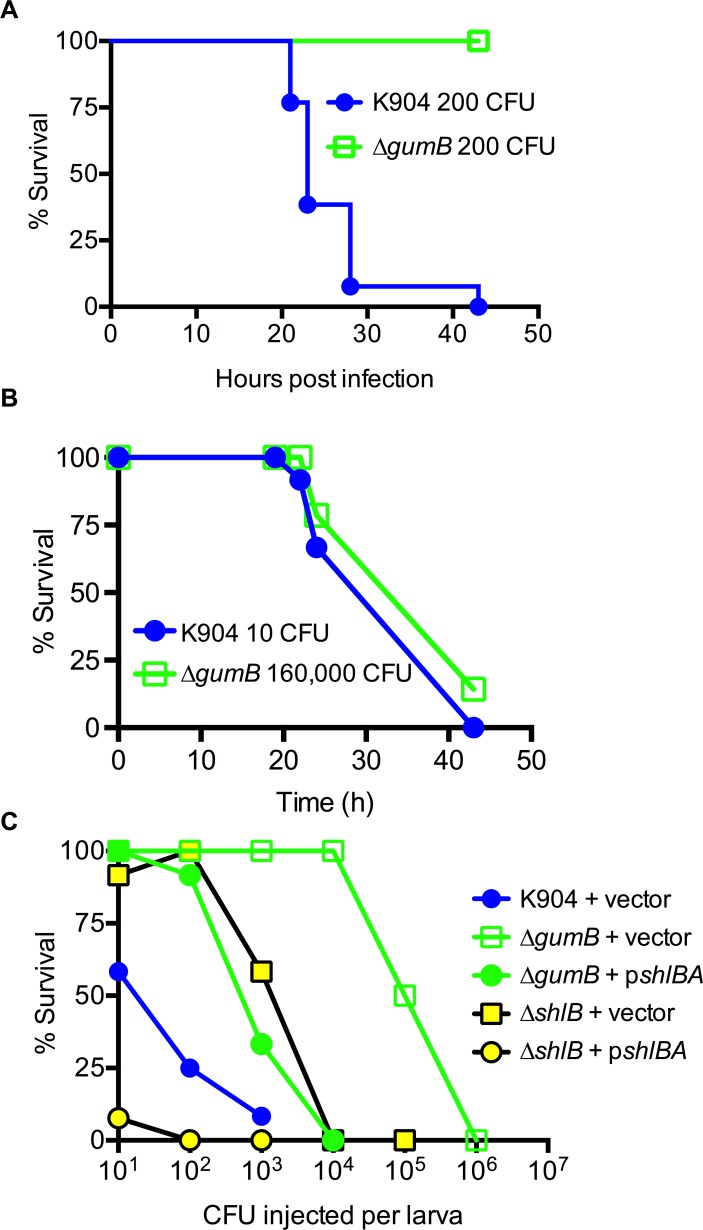
GumB is necessary for virulence in a *Galleria mellonella* model of infection via regulation of *shlBA*. Shown are survival curves of *G*. *mellonella* larvae challenged with *S*. *marcescens*. (A) *G*. *mellonella* survival over time following injection with 200 CFU is shown (n = 12 for K904, n = 13 for Δ*gumB*); p<0.001 Log-rank test. (B) Similar survival curves for *G*. *mellonella* over time were observed after treatment with either K904 or Δ*gumB* despite large differences in CFUs injected (n = 14). (C) Survival of larvae injected with the Δ*gumB* mutant and Δ*shlB* plasmid with various plasmids as indicated (n = ≥12). Vector = pMQ125; p*shlBA* = pMQ541.

Whereas the ShlA cytolysin is a known *S*. *marcescens* virulence determinant in several pathogenesis models [41–43], the role of GumB is unreported. A *Galleria mellonella* model of infection was used to test whether GumB is necessary for infection *in vivo*. When survival was analyzed over time (Fig 8A), larvae that had been injected with *S*. *marcescens* strain K904 (200 CFU/larva) started to die just after 20 h post injection. The larvae had a median survival of 23 h, and all larvae were dead by 44 h (Fig 8A).

Strikingly, the Δ*gumB* mutant strain-injected larvae (200 CFU/larva) were fully viable at 44 h when the experiment reached its endpoint (Fig 8A, p<0.001 Log-rank Test).

In a separate experiment, different doses of *S*. *marcescens* (strain K904) and the Δ*gumB* mutant strain were injected into *G*. *mellonella* larvae. A similar survival curve was observed between the two strains with 160,000 CFU of the Δ*gumB* strain (n = 14) and 10 CFU of the wild-type K904 strain (Fig 8B), which sharply delineates the >10,000-fold difference in the ability of the isogenic strains to kill a host organism.

The Δ*gumB* strain with *shlBA* constitutively expressed on a plasmid was used to test whether reduced *shlBA* expression contributes to the lack of virulence exhibited by the Δ*gumB* mutant strain in the *G*. *mellonella* pathogenesis model. Plasmid-based expression of *shlBA* increased virulence of the Δ*gumB* mutant strain compared to the Δ*gumB* mutant strain carrying the vector negative control; however, it did not confer wild-type levels of virulence (Fig 8C). The isogenic Δ*shlB* mutant was also defective in virulence compared to the wild type strain and was fully complemented with *shlBA* expressed from a plasmid. Together these results demonstrated that the *gumB* mutant is attenuated in virulence relative to the wild type, and suggest that the virulence defect is at least partially due to a loss of ShlA production.

**Fig 9:**
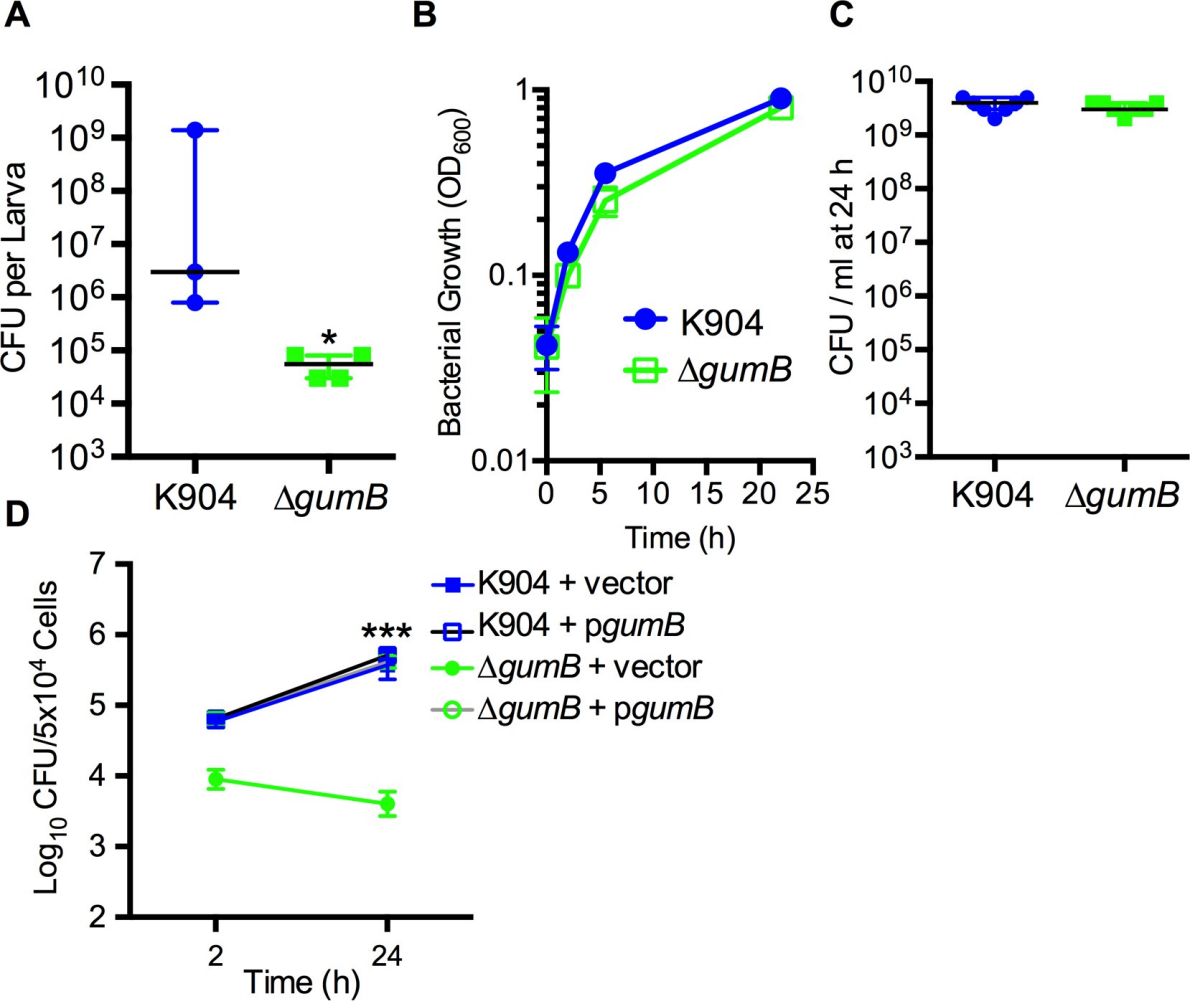
GumB is required for survival and proliferation in *G*. *mellonella* and in murine phagocytic cells. (A) Enumeration of *S*. *marcescens* K904 wild type and Δ*gumB* mutant strains 24 h after injection of 10^3^ CFU into *G*. *mellonella*. Median and range are shown, n≥3. * indicates significant difference between medians, Mann-Whitney test (p = 0.0286). (B) Growth of K904 and the Δ*gumB* mutant in *G*. *mellonella* homogenates (n = 10). Error bars indicate standard deviation. (C) Enumeration of *S*. *marcescens* K904 wild type and Δ*gumB* mutant strains 24 h growth in heat-treated *G*. *mellonella* homogenates, n = 7. Median and interquartile range is shown. (D) Representative experiment describing uptake and proliferation of *S*. *marcescens* K904 wild type and Δ*gumB* mutant strains in RAW macrophage cells (n = 3), mean and standard deviations are shown. *** indicates significant difference by 2-way ANOVA with Tukey’s post-test (p<0.001).

The mechanism for the loss of viability in the *G*. *mellonella* model was further analyzed. Bacteria were isolated from larvae before larval death (24 h post-injection), and the CFU were enumerated. There was a ~50-fold reduction in the median CFU of *S*. *marcescens* Δ*gumB* strain CFU isolated from the larvae compared to the K904 wild-type strain (p = 0.029, Mann Whitney test) (Fig 9A). To test whether the Δ*gumB* mutant strain was less capable of growth on the nutrients available in the larvae, we assessed bacterial growth in inactivated larval homogenates (Fig 9B). The lysates were heat treated to prevent melanization and growth of endogenous bacteria and clarified by centrifugation. The growth rate as assessed by optical density measurement of the Δ*gumB* mutant and wild type strains were similar in clarified lysates (Fig 9B), and the CFU achieved at 24 h were indistinguishable (p = 0.565, Mann Whitney test), (Fig 9C). We also tested whether the Δ*gumB* mutant had reduced growth at limiting oxygen concentrations, which could explain its reduced ability to proliferate within the hemolymph of the larvae. The Δ*gumB* mutant and K904 wild-type strain exhibited qualitatively equivalent colony size on LB agar plates following growth in an anaerobic bag (S5 Fig). Likewise, the Δ*gumB* mutant strain was similarly tolerant to hydrogen peroxide. Disk diffusion tests indicated that the *gumB* mutant was no more susceptible than the wild type strain (16.7±0.7 mm diameter of growth inhibition for the wild type strain and 17.4±1.7 for the Δ*gumB* strain, p = 0.203 Student’s T-test). This suggests that the Δ*gumB* mutant is more susceptible to immune components such as phagocytizing cells in the larval hemolymph rather than being unable to grow under nutrient- or oxygen-limiting conditions or exposure to reactive oxygen species in the larvae.

To test whether there is a defect in the ability of the Δ*gumB* mutant strain to survive interaction with phagocytic immune cells, we tested the ability of the bacteria proliferate in a macrophage-like murine cell line, RAW264.7 cells. The Δ*gumB* mutant strain was taken up at a reduced rate (~6-fold lower, p<0.05 Student’s t-test) compared to the wild-type K904 strain when CFU within RAW cells was assessed after 2 h of co-culture (Fig 9D). Proliferation within the RAW cells measured at 24 h post-inoculation was also measured. Whereas the wild-type CFU increased almost 7-fold within the RAW cells, there was a ~50% reduction in Δ*gumB* strain CFU (Fig 9D). Wild-type *gumB* expression from a plasmid was able to complement these defects in uptake and intracellular proliferation/survival of the *gumB* mutant, but had no effect on the wild-type K904 strain, as expected (Fig 9D).

We evaluated whether the *gumB* mutant defect in proliferation within RAW cells was due to reduced *shlBA* gene expression by expressing *shlBA* with the *nptII* promoter from a plasmid in the *gumB* mutant. We observed a significant, although partial, restoration in bacterial proliferation in RAW cells when *shlBA* was expressed in the *gumB* mutant (S8 Fig). Together, these data indicate the GumB is required for virulence and indicate that *gumB* is required for resistance to phagocytic cell responses of the innate immune system.

## Discussion

We report a T5SS-dependent, T3SS-independent mechanism by which Gram-negative bacteria can induce massive morphological changes and cellular death in human cells. The purpose of this study was to characterize and gain mechanistic insight into how Enterobacteriaceae damage the corneal epithelium, a barrier that they must overcome to gain access to the corneal stroma, a niche where they can rapidly replicate. We observed that these bacteria induce formation of blebs in human corneal epithelial cells. *S*. *marcescens*-induced blebbing was evident in several types of mammalian epithelial cells and by >30 *S*. *marcescens* isolates (100% of isolates tested), which indicates that the effect is broadly conserved and not limited to only a few bacterial isolates or mammalian cell types. The induction of blebs on intact corneas following exposure to bacteria-coated contact lenses implies that contact lens delivery of bacteria or ShlA-like cytolysins may cause damage to the ocular epithelium, possibly whether the bacteria are alive or not. Consistently, approximately 10% of contact lens wearers have an adverse contact lens wear event every year, such as red eye and irritation, and bacteria such as *S*. *marcescens* are common contaminants of contact lens cases and lenses [44–46]. Even more important is that contact lenses are a major risk factor for the vision threatening infection, microbial keratitis, with a third to a half of keratitis patients being contact lens users [18, 47].

Notably, the epithelial cell blebs reported here were similar in morphology to those *P*. *aeruginosa*-induced blebs described by Fleiszig and colleagues [6, 7, 33]. One difference between these studies and ours is that *P*. *aeruginosa* bacteria actively proliferate within the blebs [6, 7, 48], whereas *S*. *marcescens* strain K904 was not observed within the epithelial cells. Additionally, the frequency of bleb formation was higher for *S*. *marcescens*-exposed cells, with 15–20% of corneal cells exhibiting blebs after treatment with *P*. *aeruginosa* at MOI = 100, compared to up to 70% for *S*. *marcescens*-exposed cells at MOI = 200, and 26% at MOI = 50. A further difference between *P*. *aeruginosa* and *S*. *marcescens* is that bleb induction by *P*. *aeruginosa* requires a T3SS, a nanomachine largely absent in *S*. *marcescens* isolates. This study, rather, demonstrates that *S*. *marcescens* requires a T5SS to induce epithelial blebs and cytotoxicity. A subset of T3SS-lacking *P*. *aeruginosa* isolates have been described that express the T5SS secreted ExlA toxin [49, 50], and we speculate that these ExlA positive isolates may also induce blebbing and cellular death in a similar manner to *P*. *mirabilis* and *S*. *marcescens*.

Genetic analysis implicated both the T5SS composed of ShlA and ShlB and the Rcs system regulator GumB in bleb induction, control of toxicity, and facilitation of virulence. Importantly, expression of *shlBA* in non-pathogenic *E*. *coli* strains conferred the ability to induce blebs and kill corneal cells *in vitro*. This result suggests that this single virulence determinant was sufficient for the observed host-pathogen interactions, a result that was corroborated using partially purified ShlA. Furthermore, the ability of HpmA from *P*. *mirabilis* to induce blebs and kill corneal cells supports the model that this widely conserved family of cytolysins are sufficient to cause bleb formation and cell death. Indeed, the other bacteria tested in this study able to cause blebs were from species known to harbor ShlBA-like cytolysins such as *E*. *tarda* [51], although we did not prove that the strain we used has this gene. The *P*. *aeruginosa* isolate that induced blebbing has not been molecularly characterized, so it is not clear whether it induces blebbing through a T3SS or T5SS mechanism. Notably, ShlA-like proteins are found in a variety of organisms beyond those discussed above, including *Chromobacterium violaceum*, *Haemophilus ducreyi*, *Photorhabdus luminescens*, and *Yersinia* species [24, 52].

Interestingly, bacteria known to make other types of cytolysins / hemolysins such as *S*. *aureus* and *S*. *pneumoniae* did not induce bleb formation under our tested conditions, even using strains known to express the respective hemolysins, e.g. *S*. *aureus* 8325–4 [53]. Bleb formation by *Streptococcus* species may be expected since purified streptolysin O has been a key tool in understanding the biology underlying bleb formation [11, 54]. Our observations therefore suggest that many hemolytic bacteria do not generate sufficient pore forming toxins under the tested conditions to induce bleb formation in corneal cells or that the tested cells lack receptors required by the respective toxins. Furthermore, non-pore forming toxins can induce blebs. These include T5SS secreted serine proteases from *E*. *coli*, ExpC and Pet, which cause damage and induce bleb formation when added exogenously to epithelial cells [55–57].

This study implicates GumB as a *S*. *marcescens* virulence factor and mediator of host-pathogen interactions. GumB was found to be necessary for bleb induction and cytotoxicity by *S*. *marcescens* to corneal cells and virulence in *G*. *mellonella*. The bleb formation and cytotoxicity phenotypes were complemented by the wild-type *gumB* gene and several other IgaA-family genes on plasmids. This result implies that GumB is functionally conserved with other IgaA-family proteins, with the possible exception of UmoB from *P*. *mirabilis*. Several independent clones of *umoB* from different *P*. *mirabilis* genomes and different plasmid replicons were tested, suggesting that the lack of complementation was not due to a faulty complementation plasmid. The *umoB* gene codes for a protein that is more distantly related to GumB than the other tested proteins: 42% identity to GumB compared to ≥54% identity for the other tested proteins [25]. These structural differences may account for its inability to complement the Δ*gumB* mutation.

Since expression of *gumB* in *E*. *coli* was insufficient to induce blebbing in mammalian cells, and it is unlikely that an IgaA-family protein could itself cause damage to mammalian cells, we tested the hypothesis that GumB is required for bleb formation and cytotoxicity through activation of *shlBA* expression. In support of this model, *shlA* expression was highly reduced in the Δ*gumB* mutant strain and ectopic expression of *shlBA* restored the ability of the Δ*gumB* mutant to induce blebs and kill cells. These data support the model that GumB is defective in bleb induction because it fails to produce sufficient levels of the ShlA cytolysin.

Because IgaA-family proteins, similar to GumB, regulate the envelope stress response Rcs system [29, 30], we tested whether GumB functions through control of the Rcs system. Multicopy expression of *rcsC* in the wild type strain phenocopied *gumB* mutant strain phenotypes, conferring the loss of cytotoxicity and bleb induction. Additionally, mutation of *rcsB* in the *gumB* mutant strain restored bleb formation and cytotoxicity phenotypes. These were the expected outcomes if GumB functions to repress Rcs system function and RcsB inhibits *shlBA* transcription. Together these experiments support the model that GumB regulates *shlBA* expression indirectly through the Rcs system (Figs 6 and S6). Consistent with this model, the Vescovi group showed that *shlBA* transcription is directly inhibited by RcsB in *S*. *marcescens* strain RM66262, and it was proposed that phosphorylated RcsB binds to the promoters of and directly represses both *shlBA* and *flhDC* transcription [58]. Since FlhDC is a positive and direct regulator of *shlBA* expression, and activated RcsB shuts down *flhDC* expression, it is clear that RcsB can shut down *shlBA* transcription both directly and indirectly [58]. It was reported that GumB is necessary for *flhDC* expression [25] and in this study, for *shlBA*. This is in agreement with a study by DeVenanzio [58] regarding RcsB control of *shlBA*, and further supports a role for GumB in Rcs system control.

With respect to virulence, the Δ*gumB* mutant strain was highly attenuated, as injection of >10,000-fold more Δ*gumB* than wild-type K904 CFU into the larvae was required to produce similar survival profiles. The Δ*gumB* strain larvae killing defect was partially restored by multicopy expression of *shlBA* from a plasmid. Data here demonstrated that *shlBA* is essential for virulence in a *G*. *mellonella* model of infection, extending the host-range in which ShlA is a virulence factor. However, the Δ*shlB* mutant defect was not as severe as the Δ*gumB* defect, with 100-fold more CFU of the Δ*gumB* strain than Δ*shlB* strain required for complete killing of the larvae (Fig 8C). Together, these data suggested that the lack of *shlBA* expression by the *gumB* mutant is partially, but not completely responsible for reduced virulence. In addition to ShlA, other factors regulated by GumB likely contribute to virulence. These could include as metalloproteases [59, 60], FlhDC controlled phospholipase A [61], flagella [62], the hemolytic surfactant serratamolide [63], the biologically active pigment prodigiosin [64], and Rcs system regulated outer membrane vesicle [65] and capsular polysaccharide production [66]. Regardless, GumB-mediated regulation of *shlBA* accounts for a large portion of *S*. *marcescens* virulence activity.

Experiments demonstrated that GumB is necessary for replication within *G*. *mellonella*, but the Δ*gumB* mutant strain is perfectly able to use *G*. *mellonella* as a growth substrate. Experiments with the RAW macrophage-like cell line indicated that GumB is required for survival and proliferation after being phagocytized. This result suggests that GumB-regulated factors are required for the bacteria to survive within cells. In support of this notion, there is a growing body of evidence indicating a key role for ShlA in *S*. *marcescens* survival within and egress from intracellular vacuoles and regulation of autophagic processes [58, 67–69]. This is somewhat antagonistic to data from *E*. *coli* and *S*. *enterica*, where partial function alleles of *igaA* and *yrfF* increased survival of phagocytized bacteria [70–72]. The different requirements for GumB may be due to fundamental differences in the role of IgaA-family proteins between species. Alternatively, because the *yrfF* and *igaA* genes are essential for growth, different results may have resulted from the partial function of the *igaA* and *yrfF* alleles used in the previous studies [70–72].

Cellular blebs are generally a sign of impending cellular death, and in this case Enterobacteriaceae that cause contact lens associated keratitis may use this mechanism to damage the corneal epithelium, a key barrier to ocular infections [73]. We speculate that contact lens wear can facilitate contact between bacteria with ShlA-like T5SS and the ocular surface, and that even if the bacteria are killed by cleaning solutions, their surface associated and extracellularly secreted pore-forming toxins of the ShlA family could damage the epithelium. Beyond the eye, *S*. *marcescens* causes many types of nosocomial infections [15], and has been implicated in the dysbiosis associated with inflammatory diseases of the human gut [74]. In line with this observation, a recent study has shown that ShlA can cause severe damage to the digestive tract in a *Drosophila melanogaster* model [42]. ShlA also damages lung tissue and is required for hemorrhagic pneumonia, lung dysfunction, and necroptosis of epithelial cells in animal lung infection models [41, 75].

An additional observation of note was that genetic data noted here suggest that the Rcs system is a regulator of the biologically active red prodigiosin pigment, characteristic to many biotypes of *S*. *marcescens*. This conclusion was based upon multicopy expression of *rcsC* conferring a loss of pigmentation, and mutation of *rcsB* suppressing the *gumB* mutant strain pigment defect. Further studies will be required to fully analyze the role of the Rcs system in pigment regulation; however, our current data suggests a model wherein the Rcs system inhibits pigmentation under stressful conditions. This is consistent with a previous study indicating that the alarmone cAMP is used to inhibit prodigiosin biosynthesis under metabolic stress [76].

In conclusion, this study identifies a novel mechanism by which bacteria cause dramatic and lethal morphological changes in host epithelial cells to potentiate pathogenesis on mucosal surfaces, as well as the regulatory pathways underlying this important virulence activity. This ShlB and ShlA-dependent mechanism is highly toxic and employed by a broad range of Gram-negative bacterial pathogens. In the context of bacterial keratitis, this T5SS may enable bacteria to rapidly kill surface epithelial cells, allowing them to penetrate into the corneal stroma, a tissue more permissive to bacterial growth. These findings therefore implicate novel strategies for therapeutic development to prevent this conserved system from causing tissue damage and augmenting disease.

## Methods

### Ethics statement

De-identified corneas from organ donors were obtained from the Center for Organ Recovery and Education (Pittsburgh, PA) or the National Disease Research Interchange (Philadelphia, PA). Research using de-identified tissue from non-living individuals is not considered human subject research under DHHS regulation 45CFR46, and the use of decedent tissue for this project was approved by the University of Pittsburgh Committee for Oversight of Research and Clinical Training Involving Decedents.

### Microbial strains, media, and growth

*S*. *marcescens* and *P*. *mirabilis* strains are listed in Table 1. Bacteria were grown with on a TC-7 tissue culture roller (New Brunswick) in Lysogeny Broth (LB) medium [77] (0.5% yeast extract, 1% tryptone, 0.5% NaCl) with or without 1.5% agar or in M9 minimal medium [78] supplemented with glucose (0.4%) and casein amino acids (0.06%). *Escherichia coli* strains used were S17-1 λ-*pir* [79], WM3064 [80], Top10 (Invitrogen), and EC100D *pir-116* (Epicentre). *Saccharomyces cerevisiae* strain InvSc1 (Invitrogen) was grown with either YPD or SC-uracil media [81]. Antibiotics used in this study include gentamicin (10 μg ml^-1^), kanamycin (100 μg ml^-1^), and tetracycline (10 μg ml^-1^). For growth under oxygen limiting conditions, bacteria were grown on LB plates in a GAS PAK-EZ anaerobe pouch system with indicator (Becton, Dickinson and Company), and incubated at 30°C for 20 h.

**Table 1:** *P*. *mirabilis* and *S*. *marcescens* strains used in this study.

Strain	Description	Source or reference
*P*. *mirabilis*		
K2644	clinical keratitis isolate	Charles T. Campbell Laboratory
K2675	clinical keratitis isolate	Charles T. Campbell Laboratory
*S*. *marcescens*		
K904	contact lens associated keratitis isolate	[76]
CMS2265	K904 *gumB*::tn (pSC189-derived)	[25]
CMS4001	K904 Δ*gumB*	[25]
CMS4281	K904 *gumB*::tn (pBT20-derived)	This study
CMS4320	K904 *gumB*::tn (pSC189-derived)	This study
CMS4306	K904 *shlB*::tn (pSC189-derived)	This study
CMS4307	K904 *shlB*::tn (pSC189-derived)	This study
CMS4334	K904 *shlA*::tn (pSC189-derived)	This study
CMS4441	K904 Δ*gumB rscB*::pMQ118	This study
CMS4236	K904 Δ*shlB*	This study

### Cell culture, bleb formation, cell staining, and cytotoxicity assays

HCLE cells (originally from the Gipson laboratory, Harvard University) [82] were grown in monolayers as previously described [83] in 12 well MatTek glass bottomed dishes (product number P12G-1.5.14-F) that were treated with poly-L-lysine or in tissue culture treated polystyrene 12 well dishes (Costar catalog no. 3513). Cells were grown to confluence in keratinocyte serum-free medium (KSFM) (Gibco cataolog number 10724–011) supplemented with bovine pituitary extract (25 μg/ml) and epidermal growth factor (0.2 ng/ml). Bacteria were grown overnight with aeration at 30°C, washed with phosphate buffered saline (PBS), adjusted to the proper MOI in KSFM in a total volume of 1.5 ml and applied to the MatTek plate. After 2 h of incubation at 37°C with 5% CO_2_, bacteria were removed by washing cells three times with 37°C PBS, and cells covered in KSFM with or without Calcein AM (0.5 μM, ThermoFisher) for 15 minutes or CellMask plasma membrane stain (100 μM, ThermoFisher), then washed with KSFM and imaged.

Cytotoxicity assays were performed as previously described using the Presto Blue viability assay (ThermoFisher) using bacteria at the described MOI [59].

Primary epithelial cells were obtained using reagents from Gibco and Sigma Aldrich and following the protocol of Chen and colleagues [84] with some modifications. Corneal tissue obtained as noted above from the Center for Organ Recovery and Education (Pittsburgh, PA) was washed three times with Hank’s balanced salt solution supplemented with gentamycin 50μg/ml and amphotericin B 1.25μg/ml. Corneal cells were removed and digested for ~16 h at 4°C using 10 mg/ml dispase II in MESCM (a 1:1 ratio of Dulbecco’s modified Eagle medium and Ham’s F12 medium supplemented with insulin transferrin selenium solution, basic fibroblast growth factor 4 ng/ml, human leukemia inhibitory factor 10 ng/ml, gentamicin 50μg/ml and amphotericin B 1.25μg/ml). Epithelial sheets were removed and incubated with TrypLE protease mixture, neutralized with minimum essential medium supplemented with 20% FBS, and cells were plated into a 12 well plate (~8x10^5^ cells/well).

RAW 264.7 cells were grown and used as previously described [85] using kanamycin protection assays [86] to analyze bacterial proliferation.

### Porcine cornea organ culture contact lens model

Porcine corneas were purchased from Sierra Medical (Whittier, CA) and processed as previously described [87]. Corneas and adjacent scleral tissues (~ 3 mm) were excised from eyes (n = 2 per treatment group), rinsed in PBS and placed on supports composed of minimal essential medium (MEM, Gibco), rat tail collagen (1 mg/ml, Sigma), and agarose (1% w/v) in 12 well dishes. MEM was added to cover the tissue up to the limbus. Contact lenses (Air Optix Night and Day Aqua) were incubated in PBS or PBS with *S*. *marcescens* strain K904 (OD_600_ = 1.0; ~2x10^9^ CFU/ml) for 30 minutes and rinsed 2x in PBS to remove unattached bacteria, leaving ~ 1x10^8^ CFU per lens. The control and bacteria-laden lenses were applied to the corneas and together were incubated at 37°C with 5% CO_2_ for 2.5 h. Lenses were removed and the corneas fixed with glutaraldehyde (2.5%) for 20 h. Corneas were washed with PBS and post-fixed using aqueous osmium tetroxide (1%). The samples were dehydrated using increasing concentrations of ethanol (30%-100%), immersed in hexamethyldisilazane, air dried, and sputter coated with 6 nm of gold/palladium. Corneas were imaged using a JEOL JSM-6335F scanning electron microscope at 3 kV with the secondary electron imaging detector.

#### Mutagenesis and plasmid construction

Transposons were introduced into *S*. *marcescens* by conjugation as previously described [88] using a *Himar-1* based plasposon delivery plasmid pSC189 [89]. Tetracycline (10 μg ml^-1^) was used to select against donor *E*. *coli* growth, and kanamycin (100 μg ml^-1^) was used to select for *S*. *marcescens* with transposon mutations. Transposon insertions were mapped as previously described [89–91].

Cloning was performed using yeast-based recombineering of PCR generated amplicons [92, 93]. PCR amplicons used for cloning were generated using high-fidelity polymerase, Phusion (New England Biolabs) or PrimeSTAR (Clonetech). Clones were analyzed by diagnostic PCR and verified by DNA sequencing (University of Pittsburgh Genomic Research Core). Plasmids are listed in S1 Table. Directed mutagenesis was achieved by two-step allelic replacement or insertional mutagenesis as noted in the text and previously described [92, 93]. Mutations were verified using PCR outside of the cloned region on the mutagenesis plasmid.

Allelic replacement of *shlB*: To generate the *shlB* deletion strain, we cloned 698 base pairs upstream of *shlB* and 604 base pairs downstream of *shlB* using primer pairs 2619 and 2620 and 2621 and 2622, respectively, into an allelic replacement vector, pMQ460 [91], to generate pMQ473. All primer sequences are listed in S2 Table. The pMQ473 plasmid was introduced into *S*. *marcescens* strains K904 [94], PIC3611 [88], and Db11 [28]. Followed by I-SceI expression vector pMQ337 [92] to facilitate recombination. Sucrose resistant isolates were obtained on selective plates (0.5% yeast extract, 1% tryptone, 5.0% sucrose), and mutations were analyzed by PCR.

The *shlBA* operon was cloned from strain K904 using primers 3464 and 3465 and placed under the control of the *E*. *coli P*_*BAD*_ promoter on plasmid pMQ125 [92] to generate pMQ492. L-arabinose at 0.2% was used to induce expression of *shlBA* in this study. The pMQ492 plasmid was modified for constitutive expression of *shlBA* by replacing the *P*_*BAD*_ promoter with the *E*. *coli nptII* from pSC189 promoter to make pMQ541 (primers 3698 and 3699).

The *shlA* gene was mutagenized using pMQ524, a *Himar*-1 ‘*phoA* delivery plasmid described below. *E*. *coli* strain EC100D *pir*-116 with pMQ492 and pMQ524 were grown for 20 h, then plasmids were harvested. The resulting plasmid DNA was used to transform *E*. *coli* strain Top10, which does not support the replication of pMQ524, and kanamycin and gentamicin were used as selection for pMQ492 in which the transposon from pMQ524 had jumped into pMQ492. Two colonies that were blue on plates supplemented with 5-bromo-4-chloro-3-indolyl phosphate (BCIP; Sigma-Aldrich) and L-arabinose were selected, and the transposon was mapped to *shlB* in one clone and *shlA* in the other. The resulting plasmids were designated pMQ590 and pMQ591, respectively.

The pMQ524 plasposon delivery plasmid was made by combining a number of amplicons using yeast recombineering as noted above. The amplicons recombined in yeast were 1) a partial *phoA* gene, without its secretion signal from *E*. *coli* strain W3110 [95] (primers 3639 and 3640), 2) yeast replication machinery and *URA3* gene from pMQ132 (primers 3647 and 3648) [92], 3) the *bla* gene, transposase C9 gene, and one inverted repeat from pSC189 [89] (primers 3651 and 3652), and 4) the kanamycin resistance gene *nptII*, *ori*R6K and other inverted repeat from pSC189 (primers 3643 and 3644). The resulting plasmid was verified by functional analysis, PCR, and sequencing of junctions.

The *rcsB* and *rcsC* and open reading frames were amplified from *S*. *marcescens* strain K904 (*rcsB*, and *rcsC*) or *umoB* from *P*. *mirabilis* strains (K2644 and K2675) (*umoB*) and placed under control of the *E*. *coli lac* promoter on pMQ132, resulting in plasmids pMQ614, pMQ615, and pMQ600, respectively. No induction was used to express these genes in *S*. *marcescens* as this species lacks a *lac* repressor gene. Primers 3688 and 3689 were used for *rcsB*, 3691 and 3692 for *rcsC*, and 3892 and 3893 for *umoB*.

The pStvZ3 promoter probe plasmid [76] was altered to have a more convenient multicloning site. The pStvZ3 plasmid was cut with *Bam*H1 and used to transform yeast along with oligonucleotides (primers 2664 and 2665) that recombine in yeast to introduce *Sal*I, *Sac*II, *Spe*I, and *Bam*HI restriction sites to pStvZ3 and generate the plasmid pMQ544 as previously described [92].

The *rscB* and *hpmA* genes were mutagenized by targeted insertional mutagenesis. Briefly, a 316 bp long internal region of the *rcsB* gene and a 621 bp region from *hpmA* were a cloned into suicide vector pMQ544 and pMQ118, respectively. The resulting *rcsB* insertion plasmid, pMQ553, and *hpmA* insertion plasmid, pMQ596, were introduced into recipient by conjugation (as noted above). Primers to amplify the internal region were 3735 and 3736 for *rcsB* and 3896 and 3897 for *hpmA*.

The *hpmA* gene was cloned from strain K2644 into pMQ132 under control of the *E*. *coli lac* promoter using primers 3900 and 3901 to make pMQ602, and the *hpmBA* operon was cloned under control of the *E*. *coli BAD* promoter using primers 3919 and 3920 to generate pMQ601.

To generate the *shlBA* deletion variant of strain PIC3611, lambda red recombineering was used as previously described [96, 97]. A broad host-range delivery plasmid for the lambda red genes was generated by cloning the recombineering machinery from pKD46 [96] using primers 3675 and 3676 into IncQ/RSF1010-based plasmid pMQ397 [98]. After introduction of pMQ538 into strain PIC3611, it was prepared for electroporation and induced with L-arabinose and transformed with 3 μg of a PCR amplicon designed to replace *shlBA* with a kanamycin resistance cassette from pKD4 [96]. Kanamycin resistant transformants were analyzed for the *shlBA* deletion using primers that analyze the novel junctions between the *S*. *marcescens* chromosome and *nptII* resistance gene.

### Transcriptional analysis

Quantitative reverse transcriptase PCR (qRT-PCR) was used to assess gene expression as previously described [91]. To prepare bacteria for RNA extraction, single colonies were inoculated into 5 ml of LB broth, and the test tubes were incubated 30°C with aeration in 5 ml. After ~16 h, cultures were diluted to OD_600_ = 0.1 in fresh LB medium, grown to OD_600_ = 0.5, subcultured to OD_600_ = 0.1 and then grown to OD_600_ = 3. RNA and cDNA was prepared and validated to not have chromosomal DNA contamination as previously described [91]. Primers were 2638 and 2639 for the 16S rDNA gene and 4150 and 4151 for *shlA* sequences.

### Partial purification of ShlA

*Escherichia coli* strain EC100D with pMQ492 (*shlB* and *shlA*) and with pMQ175 (empty vector) were grown overnight at 30°C with aeration for 16 h in LB medium supplemented with gentamicin (10μg/ml) and L-arabinose to a final concentration of 0.2% (v/v). Bacteria were removed by centrifugation and filtration (0.22 μm), and supernatants were subject to size fractionation using a 100 kD filter unit (Centricon, Millipore). Protein fractions in PBS (200 μl) were added to HCLE cells (500 μl total volume, 10.3 μM ShlA in the pMQ492 fraction) and incubated for 3 h followed by calcein AM staining and microscopic analysis.

### Microscopic analysis

To obtain micrographs, cells on glass bottomed multiwell plates (MatTek) were imaged with a 40X objective using an Olympus IX-81 inverted microscope with an FV-1000 laser scanning confocal system (Olympus) and FluoView FV10-ASW 3.1 imaging software. For live imaging, samples in MatTek dishes were viewed with a Nikon Eclipse Ti microscope equipped with a Photometrics Cascade 1K camera and a 40X 0.30 NA objective. Metamorph software was used to obtain digital images. FIJI software was used to for image analysis [99].

***Galleria mellonella* infection assays.** *G*. *mellonella* were infected as previously described [100], with the exception that *S*. *marcescens* was suspended in PBS with 10 μg/ml tetracycline. To enumerate *S*. *marcescens*, homogenates from individual larvae were generated using a tissue grinder (Corning Pyrex 7725) in PBS with tetracycline. Lysates were serial diluted and plated on LB agar supplemented with ampicillin (150 μg/ml), chloramphenicol (30 μg/ml), and tetracycline (10 μg/ml) to prevent unwanted microbial growth.

To determine bacterial growth in larval homogenates, larvae were homogenized at a ratio of 2 larvae in 1 ml of PBS. When 15 ml of homogenate was obtained, it was centrifuged at 11,000 x g for 10 minutes to clarify the supernatant. The supernatant was heated at 95°C for 60 minutes to kill microbes and prevent melanization, which obscures optical density readings. *S*. *marcescens* cultures (1 ml) grown overnight in LB were spun down (13,000 x g for 2 minutes) and washed with PBS and then adjusted to OD_600_ = 0.05 in the larval homogenate, 150 μl was added to the wells of 96 well plates and were incubated overnight at 30°C. After 20 h, CFU were determined following serial dilution as noted above.

## Supporting information

S1 Fig Effect of select bacterial species on HCLE morphology and viability. Confocal micrographs of HCLE cells images with DIC and calcein AM viability stain after exposure to bacteria for 2 h at MOI = 200, except where noted. Yellow arrows indicate blebs extending from corneal cells. (A) HCLE cells exposed to *S. marcescens* strains, including wild type strain Db11 and an isogenic *ΔgumB* mutant strain. (B). HCLE cells exposed to various bacteria, of which only *E. tarda* and *P. aeruginosa* strain K900 induce bleb formation and cytotoxicity.
(PDF)

S2 Fig *S. marcescens* induces bleb induction in an airway cell line and secretion of ShlA is sufficient for induction of bleb formation and cytotoxicity. Confocal micrographs of human epithelial cell monolayers images with DIC and fluorescent calcein AM viability stain after challenge with bacteria. Yellow arrows indicate epithelial cell blebs. (A) A549 human airway epithelial cell line exposed to *S. marcescens* wild type K904 and *ΔgumB* strains (MOI = 200) for 2 h. (B) HCLE cells exposed to *E. coli* strain Top10 (MOI = 50, for 1 h) with a control vector, the *shlBA* expression plasmid, or a version of the *shlBA* plasmid with a transposon insertion inactivating the *shlA* gene. The control vector = pMQ125; p*shlBA* = pMQ492; p*shlBA*::tn = pMQ591.
(PDF)

S3 Fig PCR analysis for *shlA* gene in ocular isolates. All tested strains, from a variety of ocular infections (conjunctivitis, endophthalmitis, and keratitis), were positive for the *shlA* gene. (A) PCR was performed with degenerate primers due to the variable sequence of the *shlA* gene. Primer sequences were (5' to 3') gcyaacccgaayggcatcasctg for primer 4722 and yggcstrcatgcygccsags for primer 4725. The predicted amplicon is 367 base pairs. Amplicons and a size standard (SS) were separated on a 0.5% TBE PAGE gel, stained with ethidium bromide, and imaged. Strain PIC3611 was used as a positive control and the same strain with a deletion of the *shlBA* operon was used as a negative control. Sequence of the PIC3611 amplicon was 100% identical to *shlA* from several strains of *S. marcescens* over 267 bp. (B) DNA quality for all strains was verified by spectrophotometry and by PCR using primers for the conserved *oxyR* gene. Shown are amplicons for PIC3611 and the isogenic ΔshlBA mutant. This data supports that the *ΔshlBA* mutant is negative for the *shlA* amplicon because the *shlA* primers are specific and not because the DNA preparation was defective.
(PDF)

S4 Fig ShlA-mediated cytotoxicity to HCLE cells. Cytotoxicity was measured using Presto Blue reagent. HCLE monolayers, incubated with bacteria at MOI = 200 (A) or 10 (B) for 2 hours, were analyzed for viability relative to cells treated with detergent (Lysis) or LB medium (Mock). Vector = pMQ125; pshlBA = pMQ541; pgumB = pMQ480.
(PDF)

S5 Fig Pigmentation and anaerobic growth of mutant strains. (A) Photographs of bacterial pigmentation on an LB plate after growth at 30°C for 24 hours shows that multicopy of expression of *rcsC* reduces pigmentation almost as severely as mutation of *gumB*. (B) Photograph depicting that the *rcsB* mutation suppresses the gumB mutant phenotype and that this can be complemented by wild-type *rcsB* on a plasmid. Reduced pigmentation of the strain with wild-type *rcsB* on a plasmid supports the model that RcsB inhibits pigment biosynthesis. (C) Images show growth of the wild-type strain K904 and the *ΔgumB* mutant (and a *ΔgumB rcsC* double mutant) on LB agar plates grown at 30°C for 24 hours in a GAS PAK-EZ anaerobe pouch system (left panel) or at ambient oxygen levels (right). The *ΔgumB* mutant produced colonies of similar size to the wild type under both conditions indicating that the *ΔgumB* mutant does not have a significant defect for growth under low oxygen conditions.
(PDF)

S6 Fig Model for regulation of *shlBA*. Genetic model for regulation of *S. marcescens* pigment and cytolysin operons. Red bars indicate negative regulation and black arrows indicate activation. Our model predicts that in response to envelope stress, GumB acts as part of the Rcs signal transduction system to modify activity of the RcsB response regulator. In addition to directly inhibiting *shlBA* expression, RcsB also inhibits expression of the *flhDC* operon, which codes for a positive transcriptional regulator of *shlBA*. Expression of the *shlBA* operon leads to secretion of ShlA. Surface associated and surface-released ShlA forms pores in mammalian cells leading to blebbing and finally necroptosis-associated cell death.
(PDF)

S7 Fig Inhibition of bleb formation by necroptosis inhibitor GWX806742X. The graph represents data from two experiments with cell counts from n≥6 fields of view (n>80 cells per treatment group). HCLE cells treated with GWX 806742X were challenged with wild-type *S. marcescens* strain K904 at MOI = 50 and after 2 h cells were imaged and bleb frequency was measured. Mean and SD are shown. ANOVA with Tukey's post-test was used and significance is shown by asterisks. * p<0.05, ** p<0.01, **** p<0.0001. Data suggests specific inhibition of necroptosis mediator MLKL reduces bleb formation.
(PDF)

S8 Fig Role of ShlA in the Δ*gumB* RAW cell proliferation phenotype. Uptake and proliferation of *S. marcescens* K904 wild type and *ΔgumB* mutant strains with the vector (pMQ132 and *shlBA* expression plasmid pMQ541) in RAW macrophage cells (n = 4), mean and standard deviations are shown. Asterisks indicate significant difference by 2-way ANOVA with Tukey’s post-test (* = p<0.05, **** = p<0.0001).
(PDF)

S1 Movie Live cell imaging of HCLE cells exposed to mock conditions. Images of HCLE cells over three h; viewed at 400X.
(AVI)

S2 Movie Live cell imaging of HCLE cells exposed to *S. marcescens* wild-type strain K904. Images of HCLE cells over three h exposed to *S. marcescens* strain K904 at MOI = 50; viewed at 400X.
(AVI)

S3 Movie Live cell imaging of HCLE cells exposed to the *S. marcescens* Δ*gumB* mutant strain. Images of HCLE cells over three h exposed to *S. marcescens* Δ*gumB* strain at MOI = 50; viewed at 400X.
(AVI)

S1 Table Plasmids used in this study. (PDF)

S2 Table DNA oligonucleotide primers used in this study. (PDF)

## References

[1] Nisan I, Wolff C, Hanski E, Rosenshine I. Interaction of enteropathogenic *Escherichia coli* with host epithelial cells. Folia Microbiol (Praha). 1998;43(3):247–52. . 9717251 10.1007/BF02818609

[2] Goosney DL, Gruenheid S, Finlay BB. Gut feelings: enteropathogenic *E*. *coli* (EPEC) interactions with the host. Annu Rev Cell Dev Biol. 2000;16:173–89. 10.1146/annurev.cellbio.16.1.173 . 11031234

[3] Hendricks MR, Bomberger JM. Who's really in control: microbial regulation of protein trafficking in the epithelium. Am J Physiol Cell Physiol. 2014;306(3):C187–97. 10.1152/ajpcell.00277.2013 24133062 PMC3919996

[4] Charras GT. A short history of blebbing. J Microsc. 2008;231(3):466–78. 10.1111/j.1365-2818.2008.02059.x . 18755002

[5] Barros LF, Kanaseki T, Sabirov R, Morishima S, Castro J, Bittner CX, et al Apoptotic and necrotic blebs in epithelial cells display similar neck diameters but different kinase dependency. Cell Death Differ. 2003;10(6):687–97. 10.1038/sj.cdd.4401236 . 12761577

[6] Angus AA, Evans DJ, Barbieri JT, Fleiszig SM. The ADP-ribosylation domain of *Pseudomonas aeruginosa* ExoS is required for membrane bleb niche formation and bacterial survival within epithelial cells. Infect Immun. 2010;78(11):4500–10. 10.1128/IAI.00417-10 20732998 PMC2976358

[7] Hritonenko V, Mun JJ, Tam C, Simon NC, Barbieri JT, Evans DJ, et al Adenylate cyclase activity of *Pseudomonas aeruginosa* ExoY can mediate bleb-niche formation in epithelial cells and contributes to virulence. Microb Pathog. 2011;51(5):305–12. 10.1016/j.micpath.2011.08.001 PubMed Central PMCID: PMCPMC3213052. 21843628 PMC3213052

[8] Broberg CA, Zhang L, Gonzalez H, Laskowski-Arce MA, Orth K. A *Vibrio* effector protein is an inositol phosphatase and disrupts host cell membrane integrity. Science. 2010;329(5999):1660–2. 10.1126/science.1192850 . 20724587

[9] Manjo G, Joris I. Apoptosis, oncosis, and necrosis: and overview of cell death. Am J Pathol. 1995;146(1):3–15. 7856735 PMC1870771

[10] Fink SL, Cookson AL. Apoptosis, pyroptosis, and necrosis: mechanistic description of dead and dying eukryotic cells. Infect Immun. 2005;73(4):1907–16. 10.1128/IAI.73.4.1907-1916.2005 15784530 PMC1087413

[11] Babiychuk EB, Monastyrskaya K, Potez S, Draeger A. Blebbing confers resistance against cell lysis. Cell Death Differ. 2011;18(1):80–9. 10.1038/cdd.2010.81 20596076 PMC3131879

[12] Hume EB, Willcox MD. Emergence of *Serratia marcescens* as an ocular surface pathogen. Arch Soc Esp Oftalmol. 2004;79(10):475–7. . 15523567

[13] Lockhart SR, Abramson MA, Beekmann SE, Gallagher G, Riedel S, Diekema DJ, et al Antimicrobial resistance among Gram-negative bacilli causing infections in intensive care unit patients in the United States between 1993 and 2004. J Clin Microbiol. 2007;45(10):3352–9. 10.1128/JCM.01284-07 . 17715376 PMC2045364

[14] Mah-Sadorra JH, Najjar DM, Rapuano CJ, Laibson PR, Cohen EJ. *Serratia* corneal ulcers: a retrospective clinical study. Cornea. 2005;24(7):793–800. . 16160494 10.1097/01.ico.0000159738.06167.88

[15] Mahlen SD. *Serratia* infections: from military experiments to current practice. Clin Microbiol Rev. 2011;24(4):755–91. Epub 2011/10/07. 24/4/755 [pii] 10.1128/CMR.00017-11 21976608 PMC3194826

[16] Merkier AK, Rodriguez MC, Togneri A, Brengi S, Osuna C, Pichel M, et al Outbreak of a cluster with epidemic behavior due to *Serratia marcescens* after colistin administration in a hospital setting. J Clin Microbiol. 2013;51(7):2295–302. Epub 2013/05/24. JCM.03280-12 [pii] 10.1128/JCM.03280-12 23698525 PMC3697717

[17] Zhou R, Zhang R, Sun Y, Platt S, Szczotka-Flynn L, Pearlman E. Innate immune regulation of *Serratia marcescens*-induced corneal inflammation and infection. Invest Ophthalmol Vis Sci. 2012;53(11):7382–8. Epub 2012/10/04. iovs.12-10238 [pii] 10.1167/iovs.12-10238 23033384 PMC3481604

[18] Keay L, Edwards K, Naduvilath T, Taylor HR, Snibson GR, Forde K, et al Microbial keratitis predisposing factors and morbidity. Ophthalmology. 2006;113(1):109–16. 10.1016/j.ophtha.2005.08.013 . 16360210

[19] Evans DJ, Fleiszig SM. Why does the healthy cornea resist *Pseudomonas aeruginosa* infection? Am J Ophthalmol. 2013;155(6):961–70 e2. 10.1016/j.ajo.2013.03.001 23601656 PMC3718454

[20] Li P, Kwok AH, Jiang J, Ran T, Xu D, Wang W, et al Comparative genome analyses of *Serratia marcescens* FS14 reveals its high antagonistic potential. PLoS One. 2015;10(4):e0123061 10.1371/journal.pone.0123061 25856195 PMC4391916

[21] Antic I, Brothers KM, Stolzer M, Lai H, Powell E, Eutsey R, et al Gene Acquisition by a Distinct Phyletic Group within *Streptococcus pneumoniae* Promotes Adhesion to the Ocular Epithelium. mSphere. 2017;2(5). 10.1128/mSphere.00213-17 29085912 PMC5656748

[22] Kowalski RP, Romanowski EG, Mah FS, Shanks RM, Gordon YJ. Topical levofloxacin 1.5% overcomes in vitro resistance in rabbit keratitis models. Acta Ophthalmol. 2010;88(4):e120–5. Epub 2010/05/12. AOS1897 [pii] 10.1111/j.1755-3768.2010.01897.x . 20456251 PMC4739651

[23] Holloway BW. Genetic recombination in *Pseudomonas aeruginosa*. J Gen Microbiol. 1955;13(3):572–81. 10.1099/00221287-13-3-572 . 13278508

[24] Hertle R. The family of *Serratia* type pore forming toxins. Curr Protein Pept Sci. 2005;6(4):313–25. . 16101433 10.2174/1389203054546370

[25] Stella NA, Brothers KM, Callaghan JD, Passerini AM, Sigindere C, Hill PJ, et al An IgaA/UmoB-family protein from *Serratia marcescens* regulates motility, capsular polysaccharide, and secondary metabolite production. Appl Environ Microbiol. 2018;84(6):pii: e02575-17. 10.1128/AEM.02575-17 . 29305504 PMC5835751

[26] Dominguez-Bernal G, Pucciarelli MG, Ramos-Morales F, Garcia-Quintanilla M, Cano DA, Casadesus J, et al Repression of the RcsC-YojN-RcsB phosphorelay by the IgaA protein is a requisite for *Salmonella* virulence. Mol Microbiol. 2004;53(5):1437–49. Epub 2004/09/25. 10.1111/j.1365-2958.2004.04213.x [pii]. . 15387821

[27] Welch RA. Pore-forming cytolysins of gram-negative bacteria. Mol Microbiol. 1991;5(3):521–8. . 2046545 10.1111/j.1365-2958.1991.tb00723.x

[28] Flyg C, Kenne K, Boman HG. Insect pathogenic properties of *Serratia marcescens*: phage-resistant mutants with a decreased resistance to *Cecropia* immunity and a decreased virulence to *Drosophila*. J Gen Microbiol. 1980;120(1):173–81. 10.1099/00221287-120-1-173 . 7012273

[29] Cano DA, Dominguez-Bernal G, Tierrez A, Garcia-Del Portillo F, Casadesus J. Regulation of capsule synthesis and cell motility in *Salmonella enterica* by the essential gene *igaA*. Genetics. 2002;162(4):1513–23. Epub 2003/01/14. 12524328 10.1093/genetics/162.4.1513 PMC1462382

[30] Morgenstein RM, Rather PN. Role of the Umo proteins and the Rcs phosphorelay in the swarming motility of the wild type and an O-antigen (*waaL*) mutant of *Proteus mirabilis*. J Bacteriol. 2012;194(3):669–76. Epub 2011/12/06. JB.06047-11 [pii] 10.1128/JB.06047-11 22139504 PMC3264082

[31] Cho SH, Szewczyk J, Pesavento C, Zietek M, Banzhaf M, Roszczenko P, et al Detecting envelope stress by monitoring beta-barrel assembly. Cell. 2014;159(7):1652–64. Epub 2014/12/20. S0092-8674(14)01522-0 [pii] 10.1016/j.cell.2014.11.045 . 25525882

[32] Wall E, Majdalani N, Gottesman S. The Complex Rcs Regulatory Cascade. Annu Rev Microbiol. 2018 10.1146/annurev-micro-090817-062640 . 29897834

[33] Jolly AL, Takawira D, Oke OO, Whiteside SA, Chang SW, Wen ER, et al *Pseudomonas aeruginosa*-induced bleb-niche formation in epithelial cells is independent of actinomyosin contraction and enhanced by loss of cystic fibrosis transmembrane-conductance regulator osmoregulatory function. MBio. 2015;6(2):e02533 10.1128/mBio.02533-14 25714715 PMC4358002

[34] LaRocca TJ, Stivison EA, Mal-Sarkar T, Hooven TA, Hod EA, Spitalnik SL, et al CD59 signaling and membrane pores drive Syk-dependent erythrocyte necroptosis. Cell Death Dis. 2015;6:e1773 10.1038/cddis.2015.135 26018734 PMC4669712

[35] Braun V, Neuss B, Ruan Y, Schiebel E, Schoffler H, Jander G. Identification of the *Serratia marcescens* hemolysin determinant by cloning into *Escherichia coli*. J Bacteriol. 1987;169(5):2113–20. 10.1128/jb.169.5.2113-2120.1987 2437098 PMC212107

[36] Burns FR, Gray RD, Wells JT, Paterson CA. The effect of a synthetic metalloproteinase inhibitor on corneal ulceration in alkali burns and *Pseudomonas* keratitis. Matrix Suppl. 1992;1:317–8. . 1480049

[37] Ratner AJ, Hippe KR, Aguilar JL, Bender MH, Nelson AL, Weiser JN. Epithelial cells are sensitive detectors of bacterial pore-forming toxins. J Biol Chem. 2006;281(18):12994–8. 10.1074/jbc.M511431200 16520379 PMC1586115

[38] Schonherr R, Hilger M, Broer S, Benz R, Braun V. Interaction of *Serratia marcescens* hemolysin (ShlA) with artificial and erythrocyte membranes. Demonstration of the formation of aqueous multistate channels. Eur J Biochem. 1994;223(2):655–63. . 8055936 10.1111/j.1432-1033.1994.tb19038.x

[39] Gonzalez-Juarbe N, Gilley RP, Hinojosa CA, Bradley KM, Kamei A, Gao G, et al Pore-Forming Toxins Induce Macrophage Necroptosis during Acute Bacterial Pneumonia. PLoS Pathog. 2015;11(12):e1005337 10.1371/journal.ppat.1005337 26659062 PMC4676650

[40] Lusthaus M, Mazkereth N, Donin N, Fishelson Z. Receptor-Interacting Protein Kinases 1 and 3, and Mixed Lineage Kinase Domain-Like Protein Are Activated by Sublytic Complement and Participate in Complement-Dependent Cytotoxicity. Front Immunol. 2018;9:306 10.3389/fimmu.2018.00306 29527209 PMC5829068

[41] Gonzalez-Juarbe N, Mares CA, Hinojosa CA, Medina JL, Cantwell A, Dube PH, et al Requirement for *Serratia marcescens* cytolysin in a murine model of hemorrhagic pneumonia. Infect Immun. 2015;83(2):614–24. 10.1128/IAI.01822-14 25422267 PMC4294263

[42] Lee KZ, Lestradet M, Socha C, Schirmeier S, Schmitz A, Spenle C, et al Enterocyte Purge and Rapid Recovery Is a Resilience Reaction of the Gut Epithelium to Pore-Forming Toxin Attack. Cell Host Microbe. 2016;20(6):716–30. 10.1016/j.chom.2016.10.010 . 27889464

[43] Lin CS, Horng JT, Yang CH, Tsai YH, Su LH, Wei CF, et al RssAB-FlhDC-ShlBA as a major pathogenesis pathway in *Serratia marcescens*. Infect Immun. 2010;78(11):4870–81. Epub 2010/08/18. IAI.00661-10 [pii] 10.1128/IAI.00661-10 20713626 PMC2976324

[44] Ahanotu EN, Ahearn DG. Association of *Pseudomonas aeruginosa* and *Serratia marcescens* with extended-wear soft contact lenses in asymptomatic patients. Clao J. 2002;28(3):157–9. . 12144237

[45] Holden BA, La Hood D, Grant T, Newton-Howes J, Baleriola-Lucas C, Willcox MD, et al Gram-negative bacteria can induce contact lens related acute red eye (CLARE) responses. Clao J. 1996;22(1):47–52. . 8835069

[46] Yung MS, Boost M, Cho P, Yap M. Microbial contamination of contact lenses and lens care accessories of soft contact lens wearers (university students) in Hong Kong. Ophthalmic Physiol Opt. 2007;27(1):11–21. Epub 2007/01/24. OPO427 [pii] 10.1111/j.1475-1313.2006.00427.x . 17239186

[47] Bourcier T, Thomas F, Borderie V, Chaumeil C, Laroche L. Bacterial keratitis: predisposing factors, clinical and microbiological review of 300 cases. Br J Ophthalmol. 2003;87:834–8. 10.1136/bjo.87.7.834 12812878 PMC1771775

[48] Heimer SR, Evans DJ, Stern ME, Barbieri JT, Yahr T, Fleiszig SM. *Pseudomonas aeruginosa* utilizes the type III secreted toxin ExoS to avoid acidified compartments within epithelial cells. PLoS One. 2013;8(9):e73111 10.1371/journal.pone.0073111 24058462 PMC3776860

[49] Elsen S, Huber P, Bouillot S, Coute Y, Fournier P, Dubois Y, et al A type III secretion negative clinical strain of *Pseudomonas aeruginosa* employs a two-partner secreted exolysin to induce hemorrhagic pneumonia. Cell Host Microbe. 2014;15(2):164–76. 10.1016/j.chom.2014.01.003 . 24528863

[50] Reboud E, Bouillot S, Patot S, Beganton B, Attree I, Huber P. Pseudomonas aeruginosa ExlA and *Serratia marcescens* ShlA trigger cadherin cleavage by promoting calcium influx and ADAM10 activation. PLoS Pathog. 2017;13(8):e1006579 10.1371/journal.ppat.1006579 28832671 PMC5584975

[51] Hirono I, Tange N, Aoki T. Iron-regulated haemolysin gene from *Edwardsiella tarda*. Mol Microbiol. 1997;24(4):851–6. . 9194711 10.1046/j.1365-2958.1997.3971760.x

[52] Hertle R. *Serratia* type pore forming toxins. Curr Protein Pept Sci. 2000;1(1):75–89. . 12369921 10.2174/1389203003381423

[53] Bayer AS, Ramos MD, Menzies BE, Yeaman MR, Shen AJ, Cheung AL. Hyperproduction of alpha-toxin by *Staphylococcus aureus* results in paradoxically reduced virulence in experimental endocarditis: a host defense role for platelet microbicidal proteins. Infect Immun. 1997;65(11):4652–60. 9353046 10.1128/iai.65.11.4652-4660.1997 PMC175667

[54] Keyel PA, Loultcheva L, Roth R, Salter RD, Watkins SC, Yokoyama WM, et al Streptolysin O clearance through sequestration into blebs that bud passively from the plasma membrane. J Cell Sci. 2011;124(Pt 14):2414–23. 10.1242/jcs.076182 21693578 PMC3124372

[55] Betancourt-Sanchez M, Navarro-Garcia F. Pet secretion, internalization and induction of cell death during infection of epithelial cells by enteroaggregative *Escherichia coli*. Microbiology. 2009;155(Pt 9):2895–906. 10.1099/mic.0.029116-0 . 19542001

[56] Navarro-Garcia F, Canizalez-Roman A, Sui BQ, Nataro JP, Azamar Y. The serine protease motif of EspC from enteropathogenic *Escherichia coli* produces epithelial damage by a mechanism different from that of Pet toxin from enteroaggregative *E*. *coli*. Infect Immun. 2004;72(6):3609–21. 10.1128/IAI.72.6.3609-3621.2004 15155671 PMC415714

[57] Navarro-Garcia F, Sears C, Eslava C, Cravioto A, Nataro JP. Cytoskeletal effects induced by pet, the serine protease enterotoxin of enteroaggregative *Escherichia coli*. Infect Immun. 1999;67(5):2184–92. 10225873 10.1128/iai.67.5.2184-2192.1999 PMC115956

[58] Di Venanzio G, Stepanenko TM, Garcia Vescovi E. *Serratia marcescens* ShlA pore-forming toxin is responsible for early induction of autophagy in host cells and is transcriptionally regulated by RcsB. Infect Immun. 2014;82(9):3542–54. 10.1128/IAI.01682-14 24914224 PMC4187834

[59] Brothers KM, Stella NA, Romanowski EG, Kowalski RP, Shanks RM. EepR mediates secreted protein production, desiccation survival, and proliferation in a corneal infection model. Infect Immun. 2015 10.1128/IAI.00466-15 . 26324535 PMC4598396

[60] Ishii K, Adachi T, Hamamoto H, Sekimizu K. *Serratia marcescens* suppresses host cellular immunity via the production of an adhesion-inhibitory factor against immunosurveillance cells. J Biol Chem. 2014;289(9):5876–88. Epub 2014/01/09. M113.544536 [pii] 10.1074/jbc.M113.544536 24398686 PMC3937657

[61] Anderson MT, Mitchell LA, Mobley HLT. Cysteine Biosynthesis Controls *Serratia marcescens* Phospholipase Activity. J Bacteriol. 2017;199(16). 10.1128/JB.00159-17 28559296 PMC5527384

[62] Wei CF, Tsai YH, Tsai SH, Lin CS, Chang CJ, Lu CC, et al Cross-talk between bacterial two-component systems drives stepwise regulation of flagellar biosynthesis in swarming development. Biochem Biophys Res Commun. 2017;489(1):70–5. 10.1016/j.bbrc.2017.05.077 . 28522292

[63] Shanks RM, Stella NA, Lahr RM, Wang S, Veverka TI, Kowalski RP, et al Serratamolide is a Hemolytic Factor Produced by *Serratia marcescens*. PLoS One. 2012;7(5):e36398 Epub 2012/05/23. 10.1371/journal.pone.0036398 [pii]. 22615766 PMC3353980

[64] Perez-Tomas R, Vinas M. New insights on the antitumoral properties of prodiginines. Curr Med Chem. 2010;17(21):2222–31. Epub 2010/05/13. BSP/CMC/E-Pub/ 136 [pii]. . 20459382 10.2174/092986710791331103

[65] McMahon KJ, Castelli ME, Garcia Vescovi E, Feldman MF. Biogenesis of outer membrane vesicles in *Serratia marcescens* is thermoregulated and can be induced by activation of the Rcs phosphorelay system. J Bacteriol. 2012;194(12):3241–9. Epub 2012/04/12. JB.00016-12 [pii] 10.1128/JB.00016-12 22493021 PMC3370869

[66] Anderson MT, Mitchell LA, Zhao L, Mobley HLT. Capsule Production and Glucose Metabolism Dictate Fitness during *Serratia marcescens* Bacteremia. MBio. 2017;8(3):e00740–17. 10.1128/mBio.00740-17 28536292 PMC5442460

[67] Hertle R, Schwarz H. *Serratia marcescens* internalization and replication in human bladder epithelial cells. BMC Infect Dis. 2004;4:16 10.1186/1471-2334-4-16 . 15189566 PMC441377

[68] Fedrigo GV, Campoy EM, Di Venanzio G, Colombo MI, Garcia Vescovi E. *Serratia marcescens* is able to survive and proliferate in autophagic-like vacuoles inside non-phagocytic cells. PLoS One. 2011;6(8):e24054 10.1371/journal.pone.0024054 21901159 PMC3162031

[69] Di Venanzio G, Lazzaro M, Morales ES, Krapf D, Garcia Vescovi E. A pore-forming toxin enables *Serratia* a nonlytic egress from host cells. Cell Microbiol. 2017;19(2). 10.1111/cmi.12656 . 27532510

[70] Van Tyne D, Ciolino JB, Wang J, Durand ML, Gilmore MS. Novel Phagocytosis-Resistant Extended-Spectrum beta-Lactamase-Producing *Escherichia coli* From Keratitis. JAMA Ophthalmol. 2016;134(11):1306–9. 10.1001/jamaophthalmol.2016.3283 27631542 PMC5106311

[71] Miskinyte M, Sousa A, Ramiro RS, de Sousa JA, Kotlinowski J, Caramalho I, et al The genetic basis of *Escherichia coli* pathoadaptation to macrophages. PLoS Pathog. 2013;9(12):e1003802 Epub 2013/12/19. 10.1371/journal.ppat.1003802 [pii]. 24348252 PMC3861542

[72] Cano DA, Martinez-Moya M, Pucciarelli MG, Groisman EA, Casadesus J, Garcia-Del Portillo F. *Salmonella enterica* serovar Typhimurium response involved in attenuation of pathogen intracellular proliferation. Infect Immun. 2001;69(10):6463–74. Epub 2001/09/13. 10.1128/IAI.69.10.6463-6474.2001 11553591 PMC98782

[73] Evans DJ, McNamara NA, Fleiszig SM. Life at the front: dissecting bacterial-host interactions at the ocular surface. Ocul Surf. 2007;5(3):213–27. . 17660895 10.1016/s1542-0124(12)70612-2

[74] Hoarau G, Mukherjee PK, Gower-Rousseu C, Hager C, Chandra J, Retueto MA, et al Bacteriome and Mycobiome Interactions Underscore Microbial Dysbiosis in Familial Crohn's Disease. Mbio. 2016;20(7):e01250–16. 10.1128/mBio.01250-16 PMC5030358 27651359

[75] Gonzalez-Juarbe N, Bradley KM, Shenoy AT, Gilley RP, Reyes LF, Hinojosa CA, et al Pore-forming toxin-mediated ion dysregulation leads to death receptor-independent necroptosis of lung epithelial cells during bacterial pneumonia. Cell Death Differ. 2017; 24(5)917–928. 10.1038/cdd.2017.49 . 28387756 PMC5423117

[76] Kalivoda EJ, Stella NA, Aston MA, Fender JE, Thompson PP, Kowalski RP, et al Cyclic AMP negatively regulates prodigiosin production by *Serratia marcescens*. Res Microbiol. 2010;161(2):158–67. Epub 2010/01/05. S0923-2508(09)00256-3 [pii] 10.1016/j.resmic.2009.12.004 20045458 PMC2846241

[77] Bertani G. Studies on lysogenesis. I. The mode of phage liberation by lysogenic *Escherichia coli*. J Bacteriol. 1951;62(3):293–300. Epub 1951/09/01. 14888646 10.1128/jb.62.3.293-300.1951 PMC386127

[78] Adams MH. Bacteriophages. New York, NY: Interscience Publishers Inc; 1959.

[79] Miller VL, Mekalanos JJ. A novel suicide vector and its use in construction of insertion mutations: osmoregulation of outer membrane proteins and virulence determinants in *Vibrio cholerae* requires *toxR*. J Bacteriol. 1988;170:2575 10.1128/jb.170.6.2575-2583.1988 2836362 PMC211174

[80] Caiazza NC, Lies DP, Newman DK. Phototrophic Fe(II) oxidation promotes organic carbon acquisition by *Rhodobacter capsulatus* SB1003. Appl Environ Microbiol. 2007;73(19):6150–8. 10.1128/AEM.02830-06 17693559 PMC2074999

[81] Burke D. DD, Stearns T. Methods In Yeast Genetics, A Cold Spring Harbor Laboratory Course Manual. Plainview, NY: Cold Harbor laboratory Press; 2000.

[82] Gipson IK, Spurr-Michaud S, Argüeso P, Tisdale A, Ng TF, Russo CL. Mucin gene expression in immortalized human conreal-limbal and conjunc tival epithelial cell lines. Invest Ophthalmol Vis Sci. 2003;44(6):2496–506. 12766048 10.1167/iovs.02-0851

[83] Shanks RM, Davra VR, Romanowski EG, Brothers KM, Stella NA, Godboley D, et al An Eye to a Kill: Using Predatory Bacteria to Control Gram-Negative Pathogens Associated with Ocular Infections. PLoS One. 2013;8(6):e66723 10.1371/journal.pone.0066723 23824756 PMC3688930

[84] Chen SY, Hayashida Y, Chen MY, Xie HT, Tseng SC. A new isolation method of human limbal progenitor cells by maintaining close association with their niche cells. Tissue Eng Part C Methods. 2011;17(5):537–48. 10.1089/ten.TEC.2010.0609 21175372 PMC3129703

[85] Horzempa J, Carlson PE Jr., O'Dee DM, Shanks RM, Nau GJ. Global transcriptional response to mammalian temperature provides new insight into *Francisella tularensis* pathogenesis. BMC Microbiol. 2008;8(1):172 10.1186/1471-2180-8-172 . 18842136 PMC2576331

[86] Brett PJ, Burtnick MN, Su H, Nair V, Gherardini FC. iNOS activity is critical for the clearance of *Burkholderia mallei* from infected RAW 264.7 murine macrophages. Cell Microbiol. 2008;10(2):487–98. 10.1111/j.1462-5822.2007.01063.x 17970762 PMC2228653

[87] Brothers KM, Stella NA, Hunt KM, Romanowski EG, Liu X, Klarlund JK, et al Putting on the brakes: Bacterial impediment of wound healing. Sci Rep. 2015;5:14003 Epub 2015/09/15. 10.1038/srep14003 26365869 PMC4650533

[88] Shanks RM, Stella NA, Kalivoda EJ, Doe MR, O'Dee DM, Lathrop KL, et al A *Serratia marcescens* OxyR homolog mediates surface attachment and biofilm formation. J Bacteriol. 2007;189(20):7262–72. Epub 2007/08/07. JB.00859-07 [pii] 10.1128/JB.00859-07 17675374 PMC2168423

[89] Chiang SL, Rubin EJ. Construction of a mariner-based transposon for epitope-tagging and genomic targeting. Gene. 2002;296(1–2):179–85. Epub 2002/10/18. S0378111902008569 [pii]. . 12383515 10.1016/s0378-1119(02)00856-9

[90] O'Toole GA, Pratt LA, Watnick PI, Newman DK, Weaver VB, Kolter R. Genetic approaches to study of biofilms. Methods Enzymol. 1999;310:91–109. Epub 1999/11/05. . 10547784 10.1016/s0076-6879(99)10008-9

[91] Stella NA, Lahr RM, Brothers KM, Kalivoda EJ, Hunt KM, Kwak DH, et al *Serratia marcescens* cyclic AMP-receptor protein controls transcription of EepR, a novel regulator of antimicrobial secondary metabolites. J Bacteriol. 2015;197(15):2468–78. Epub 2015/04/22. JB.00136-15 [pii] 10.1128/JB.00136-15 . 25897029 PMC4518835

[92] Shanks RM, Kadouri DE, MacEachran DP, O'Toole GA. New yeast recombineering tools for bacteria. Plasmid. 2009;62(2):88–97. Epub 2009/05/30. 10.1016/j.plasmid.2009.05.002 19477196 PMC2737453

[93] Shanks RM, Stella NA, Arena KE, Fender JE. Mutation of *crp* mediates *Serratia marcescens* serralysin and global secreted protein production. Res Microbiol. 2013;164(1):38–45. Epub 2012/10/18. S0923-2508(12)00151-9 [pii] 10.1016/j.resmic.2012.10.006 23072819 PMC3534799

[94] Kalivoda EJ, Stella NA, O'Dee DM, Nau GJ, Shanks RM. The cyclic AMP-dependent catabolite repression system of *Serratia marcescens* mediates biofilm formation through regulation of type 1 fimbriae. Appl Environ Microbiol. 2008;74(11):3461–70. Epub 2008/04/22. AEM.02733-07 [pii] 10.1128/AEM.02733-07 18424546 PMC2423026

[95] Bachmann BJ. Pedigrees of some mutant strains of *Escherichia coli* K-12. Bacteriol Rev. 1972;36(4):525–57. 4568763 10.1128/br.36.4.525-557.1972 PMC408331

[96] Datsenko KA, Wanner BL. One-step inactivation of chromosomal genes in *Escherichia coli* K-12 using PCR products. Proc Natl Acad Sci U S A. 2000;97(12):6640–5. 10.1073/pnas.120163297 . 10829079 PMC18686

[97] Rossi MS, Paquelin A, Ghigo JM, Wandersman C. Haemophore-mediated signal transduction across the bacterial cell envelope in *Serratia marcescens*: the inducer and the transported substrate are different molecules. Mol Microbiol. 2003;48(6):1467–80. . 12791131 10.1046/j.1365-2958.2003.03516.x

[98] Mukherjee S, Brothers KM, Shanks RMQ, Kadouri DE. Visualizing *Bdellovibrio bacteriovorus* by Using the tdTomato Fluorescent Protein. Appl Environ Microbiol. 2015;82(6):1653–61. 10.1128/AEM.03611-15 26712556 PMC4784026

[99] Schneider CA, Rasband WS, Eliceiri KW. NIH Image to ImageJ: 25 years of image analysis. Nat Methods. 2012;9(7):671–5. 10.1038/nmeth.2089 22930834 PMC5554542

[100] O'Hara JA, Ambe LA, Casella LG, Townsend BM, Pelletier MR, Ernst RK, et al Activities of vancomycin-containing regimens against colistin-resistant *Acinetobacter baumannii* clinical strains. Antimicrob Agents Chemother. 2013;57(5):2103–8. Epub 2013/02/21. AAC.02501-12 [pii] 10.1128/AAC.02501-12 23422916 PMC3632926

