# Supplementary material for: Blowing epithelial cell bubbles with GumB: ShlA-family pore-forming toxins induce blebbing and rapid cellular death in corneal epithelial cells
Source: PLoS Pathog. 2019 Jun 20;15(6):e1007825. doi: 10.1371/journal.ppat.1007825 (PMC6586354; doi:10.1371/journal.ppat.1007825)
Supplement: S1 Fig — Confocal micrographs of HCLE cells images with DIC and calcein AM viability stain after exposure to bacteria for 2 h at MOI = 200, except where noted. Yellow arrows indicate blebs extending from corneal cells. (A) HCLE cells exposed to S. marcescens strains, including wild type strain Db11 and an isogenic ΔgumB mutant strain. (B). HCLE cells exposed to various bacteria, of which only E. tarda and P. aeruginosa strain K900 induce bleb formation and cytotoxicity. (PDF) [file ppat.1007825.s001.pdf]

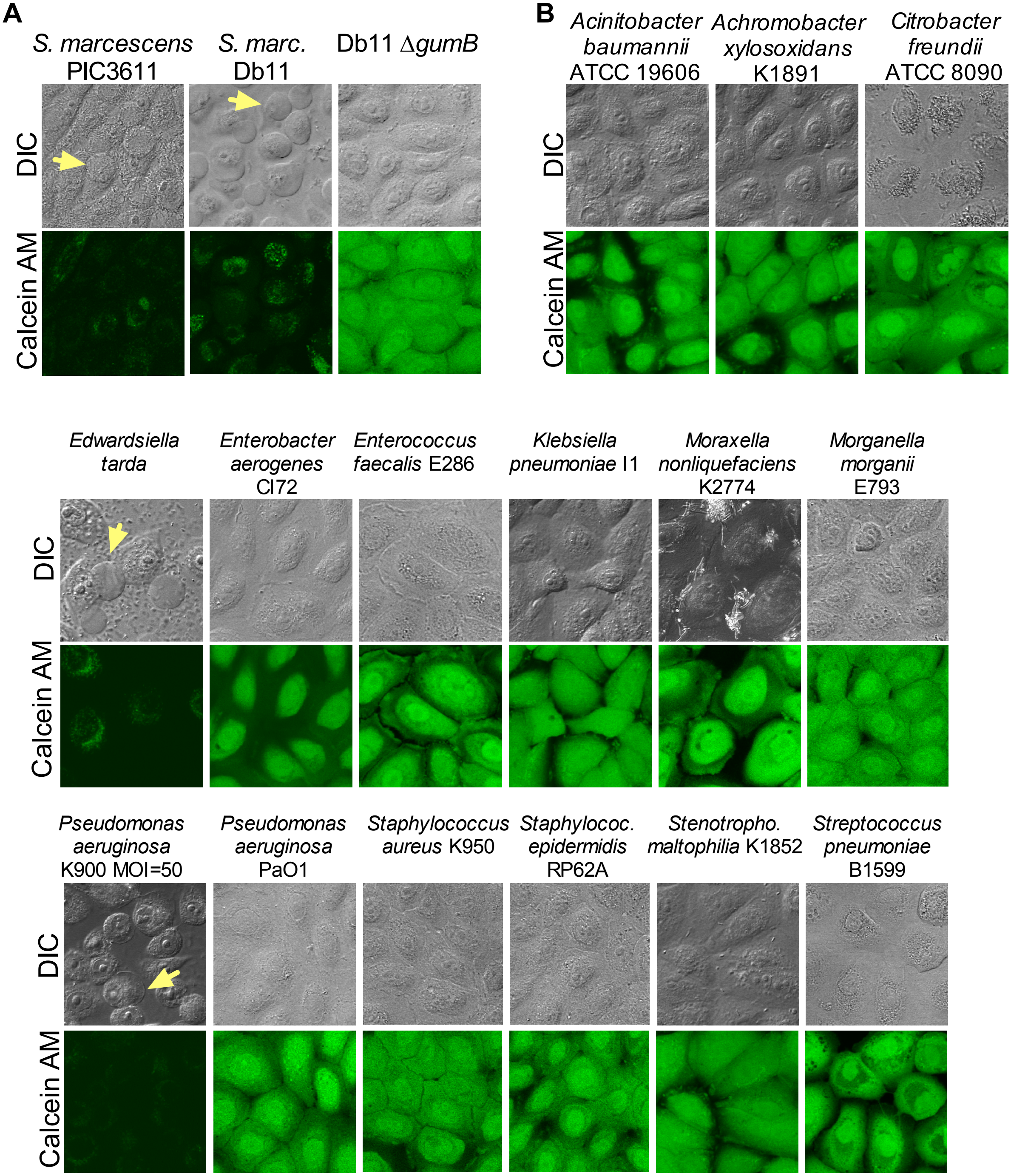

S1 Fig. Effect of select bacterial species on HCLE morphology and viability. Confocal micrographs of HCLE cells images with DIC and calcein AM viability stain after exposure to bacteria for 2 hours at MOI=200, except were noted. Yellow arrows indicate blebs extending from corneal cells. (A) HCLE cells exposed to *S. marcescens* strains including Db11 and an isogenic Δ*gumB* Mutant. (B). HCLE cells exposed to various bacteria, of which only *E. tarda* and *P. aeruginosa* strain K900 induce bleb formation.
