# Supplementary material for: Blowing epithelial cell bubbles with GumB: ShlA-family pore-forming toxins induce blebbing and rapid cellular death in corneal epithelial cells
Source: PLoS Pathog. 2019 Jun 20;15(6):e1007825. doi: 10.1371/journal.ppat.1007825 (PMC6586354; doi:10.1371/journal.ppat.1007825)
Supplement: S2 Fig — Confocal micrographs of human epithelial cell monolayers images with DIC and fluorescent calcein AM viability stain after challenge with bacteria. Yellow arrows indicate epithelial cell blebs. (A) A549 human airway epithelial cell line exposed to S. marcescens wild type K904 and ΔgumB strains (MOI = 200) for 2 h. (B) HCLE cells exposed to E. coli strain Top10 (MOI = 50, for 1 h) with a control vector, the shlBA expression plasmid, or a version of the shlBA plasmid with a transposon insertion inactivating the shlA gene. The control vector = pMQ125; pshlBA = pMQ492; pshlBA::tn = pMQ591. (PDF) [file ppat.1007825.s002.pdf]

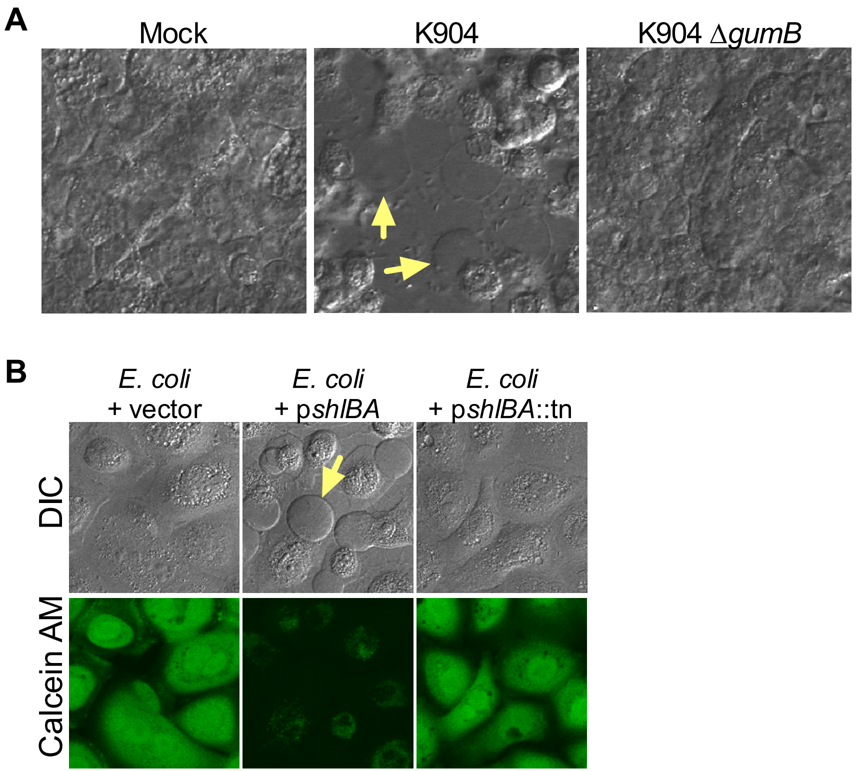

S2 Fig. *S. marcescens* induces bleb induction in an airway cell line and secretion of ShlA is sufficient for induction of bleb formation and cytotoxicity. Confocal micrographs of human epithelial cell monolayers images with DIC and fluorescent calcein AM viability stain after challenge with bacteria. Yellow arrows indicate epithelial cell blebs. (A) A549 human airway epithelial cell line exposed to *S. marcescens* wild type K904 and Δ*gumB* strains (MOI = 200) for 2 h. (B) HCLE cells exposed to *E. coli* strain Top10 (MOI = 50, for 1 h) with a control vector, the *shlBA* expression plasmid, or a version of the *shlBA* plasmid with a transposon insertion inactivating the *shlA* gene. The control vector = pMQ125; p*shlBA* = pMQ492; p*shlBA*::tn = pMQ591.
