# Supplementary material for: Blowing epithelial cell bubbles with GumB: ShlA-family pore-forming toxins induce blebbing and rapid cellular death in corneal epithelial cells
Source: PLoS Pathog. 2019 Jun 20;15(6):e1007825. doi: 10.1371/journal.ppat.1007825 (PMC6586354; doi:10.1371/journal.ppat.1007825)
Supplement: S3 Fig — All tested strains, from a variety of ocular infections (conjunctivitis, endophthalmitis, and keratitis), were positive for the shlA gene. (A) PCR was performed with degenerate primers due to the variable sequence of the shlA gene. Primer sequences were (5' to 3') gcyaacccgaayggcatcasctg for primer 4722 and yggcstrcatgcygccsags for primer 4725. The predicted amplicon is 367 base pairs. Amplicons and a size standard (SS) were separated on a 0.5% TBE PAGE gel, stained with ethidium bromide, and imaged. Strain PIC3611 was used as a positive control and the same strain with a deletion of the shlBA operon was used as a negative control. Sequence of the PIC3611 amplicon was 100% identical to shlA from several strains of S. marcescens over 267 bp. (B) DNA quality for all strains was verified by spectrophotometry and by PCR using primers for the conserved oxyR gene. Shown are amplicons for PIC3611 and the isogenic ΔshlBA mutant. This data supports that the ΔshlBA mutant is negative for the shlA amplicon because the shlA primers are specific and not because the DNA preparation was defective. (PDF) [file ppat.1007825.s003.pdf]

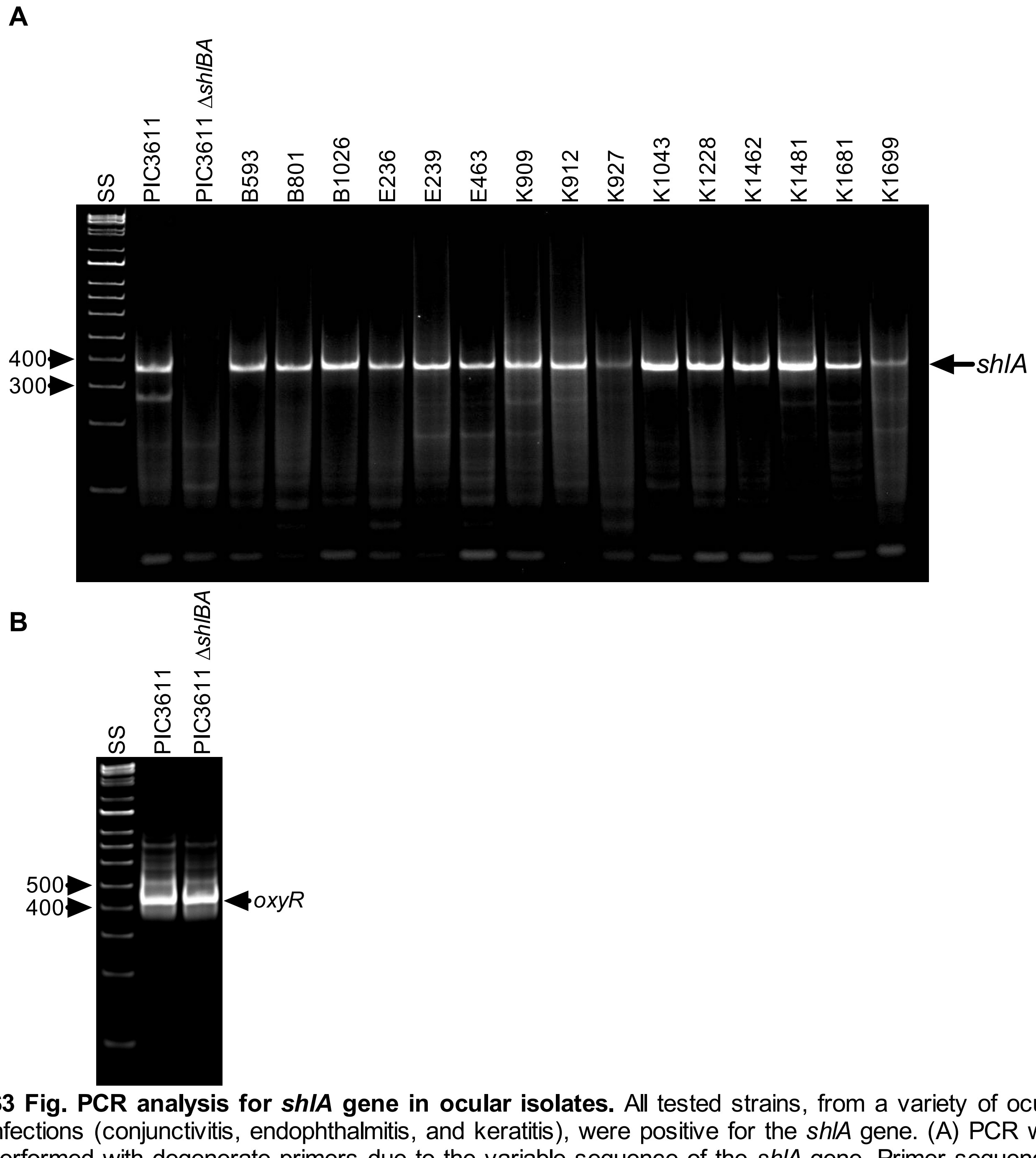

S3 Fig. PCR analysis for *shlA* gene in ocular isolates. All tested strains, from a variety of ocular infections (conjunctivitis, endophthalmitis, and keratitis), were positive for the *shlA* gene. (A) PCR was performed with degenerate primers due to the variable sequence of the *shlA* gene. Primer sequences were (5' to 3') gcyaacccgaayggcatcasctg for primer 4722 and yggcstrcatgcygccsags for primer 4725. The predicted amplicon is 367 base pairs. Amplicons and a size standard (SS) were separated on a 0.5% TBE PAGE gel, stained with ethidium bromide, and imaged. Strain PIC3611 was used as a positive control and the same strain with a deletion of the *shlBA* operon was used as a negative control. Sequence of the PIC3611 amplicon was 100% identical to *shlA* from several strains of *S. marcescens* over 267 bp. (B) DNA quality for all strains was verified by spectrophotometry and by PCR using primers for the conserved *oxyR* gene. Shown are amplicons for PIC3611 and the isogenic Δ*shlBA* mutant. This data supports that the Δ*shlBA* mutant is negative for the *shlA* amplicon because the *shlA* primers are specific and not because the DNA preparation was defective.
