# Supplementary material for: Blowing epithelial cell bubbles with GumB: ShlA-family pore-forming toxins induce blebbing and rapid cellular death in corneal epithelial cells
Source: PLoS Pathog. 2019 Jun 20;15(6):e1007825. doi: 10.1371/journal.ppat.1007825 (PMC6586354; doi:10.1371/journal.ppat.1007825)
Supplement: S4 Fig — Cytotoxicity was measured using Presto Blue reagent. HCLE monolayers, incubated with bacteria at MOI = 200 (A) or 10 (B) for 2 hours, were analyzed for viability relative to cells treated with detergent (Lysis) or LB medium (Mock). Vector = pMQ125; pshlBA = pMQ541; pgumB = pMQ480. (PDF) [file ppat.1007825.s004.pdf]

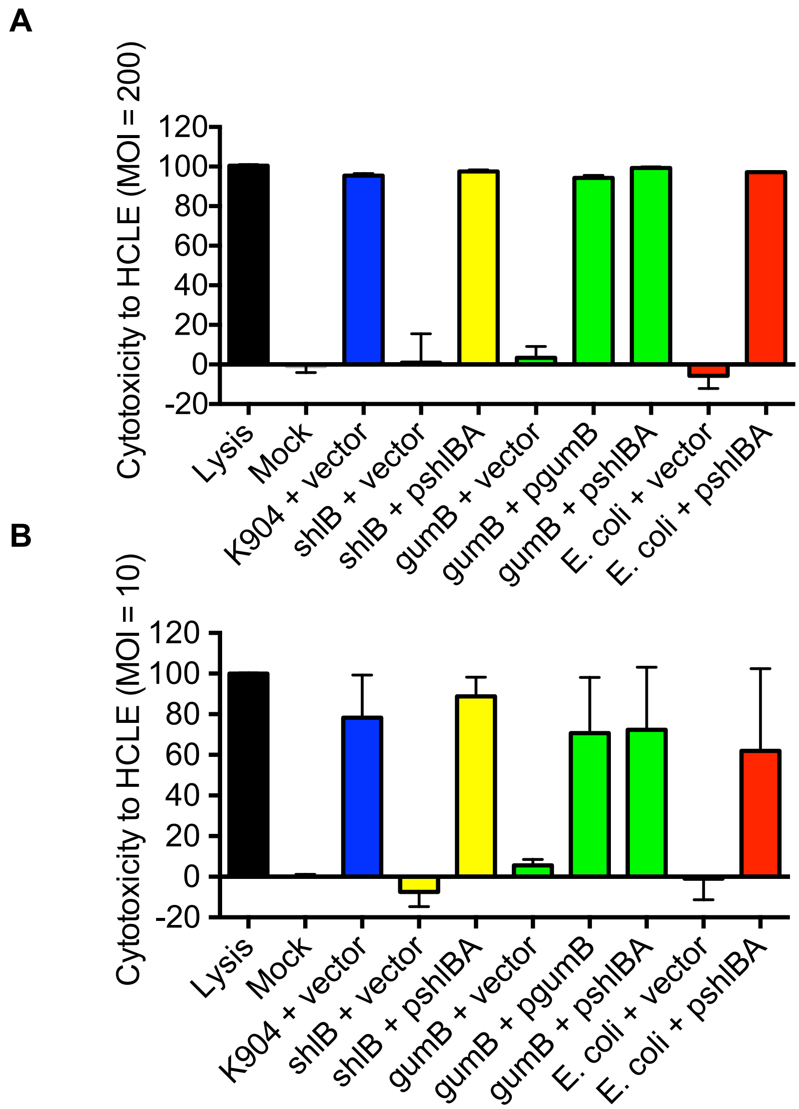

S4 Fig. ShlA-mediated cytotoxicity to HCLE cells. Cytotoxicity was measured using Presto Blue reagent. HCLE monolayers, incubated with bacteria at MOI = 200 (A) or 10 (B) for 2 hours, were analyzed for viability relative to cells treated with detergent (Lysis) or LB medium (Mock). Vector = pMQ125; p*shlBA* = pMQ541; p*gumB* = pMQ480.
