# Supplementary material for: Blowing epithelial cell bubbles with GumB: ShlA-family pore-forming toxins induce blebbing and rapid cellular death in corneal epithelial cells
Source: PLoS Pathog. 2019 Jun 20;15(6):e1007825. doi: 10.1371/journal.ppat.1007825 (PMC6586354; doi:10.1371/journal.ppat.1007825)
Supplement: S5 Fig — (A) Photographs of bacterial pigmentation on an LB plate after growth at 30°C for 24 hours shows that multicopy of expression of rcsC reduces pigmentation almost as severely as mutation of gumB. (B) Photograph depicting that the rcsB mutation suppresses the gumB mutant phenotype and that this can be complemented by wild-type rcsB on a plasmid. Reduced pigmentation of the strain with wild-type rcsB on a plasmid supports the model that RcsB inhibits pigment biosynthesis. (C) Images show growth of the wild-type strain K904 and the ΔgumB mutant (and a ΔgumB rcsC double mutant) on LB agar plates grown at 30°C for 24 hours in a GAS PAK-EZ anaerobe pouch system (left panel) or at ambient oxygen levels (right). The ΔgumB mutant produced colonies of similar size to the wild type under both conditions indicating that the ΔgumB mutant does not have a significant defect for growth under low oxygen conditions. (PDF) [file ppat.1007825.s005.pdf]

S5 Fig

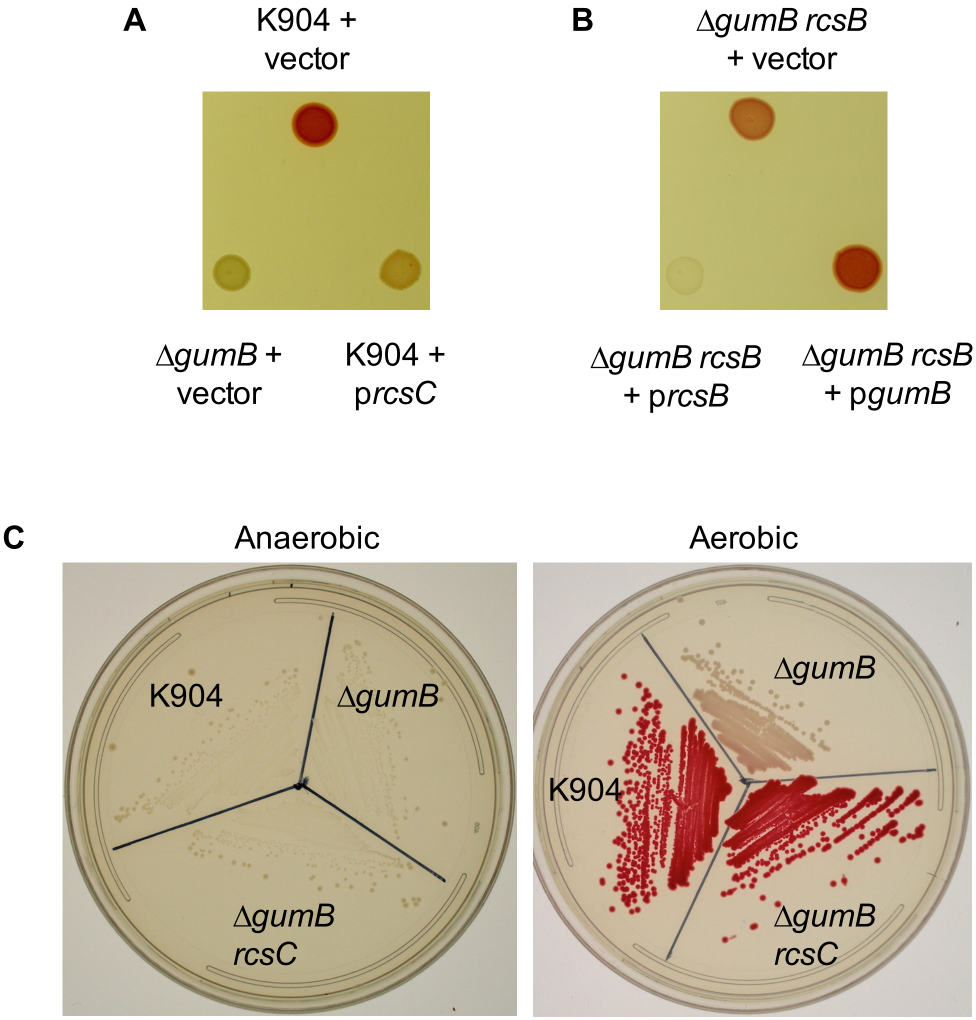

S5 Fig. Pigmentation and anaerobic growth of mutant strains. (A) Photographs of bacterial pigmentation on an LB plate after growth at 30˚C for 24 hours shows that multicopy of expression of *rcsC* reduces pigmentation almost as severly as mutation of *gumB*. (B) Photograph depicting that the *rcsB* mutation suppresses the *gumB* mutant phenotype and that this can be complemented by wild-type *rcsB* on a plasmid. Reduced pigmentation of the strain with wild-type *rcsB* on a plasmid supports the model that RcsB inhibits pigment biosynthesis. (C) Images show growth of the wild-type strain K904 and the ΔgumB mutant (and a Δ*gumB rcsC* double mutant) on LB agar plates grown at 30˚C for 24 hours in a GAS PAK-EZ anaerobe pouch system (left panel) or at ambient oxygen levels (right). The Δ*gumB* mutant produced colonies of similar size to the wild type under both conditions indicating that the Δ*gumB* mutant does not have a significant defect for growth under low oxygen conditions.
