# Supplementary material for: Blowing epithelial cell bubbles with GumB: ShlA-family pore-forming toxins induce blebbing and rapid cellular death in corneal epithelial cells
Source: PLoS Pathog. 2019 Jun 20;15(6):e1007825. doi: 10.1371/journal.ppat.1007825 (PMC6586354; doi:10.1371/journal.ppat.1007825)
Supplement: S6 Fig — Genetic model for regulation of S. marcescens pigment and cytolysin operons. Red bars indicate negative regulation and black arrows indicate activation. Our model predicts that in response to envelope stress, GumB acts as part of the Rcs signal transduction system to modify activity of the RcsB response regulator. In addition to directly inhibiting shlBA expression, RcsB also inhibits expression of the flhDC operon, which codes for a positive transcriptional regulator of shlBA. Expression of the shlBA operon leads to secretion of ShlA. Surface associated and surface-released ShlA forms pores in mammalian cells leading to blebbing and finally necroptosis-associated cell death. (PDF) [file ppat.1007825.s006.pdf]

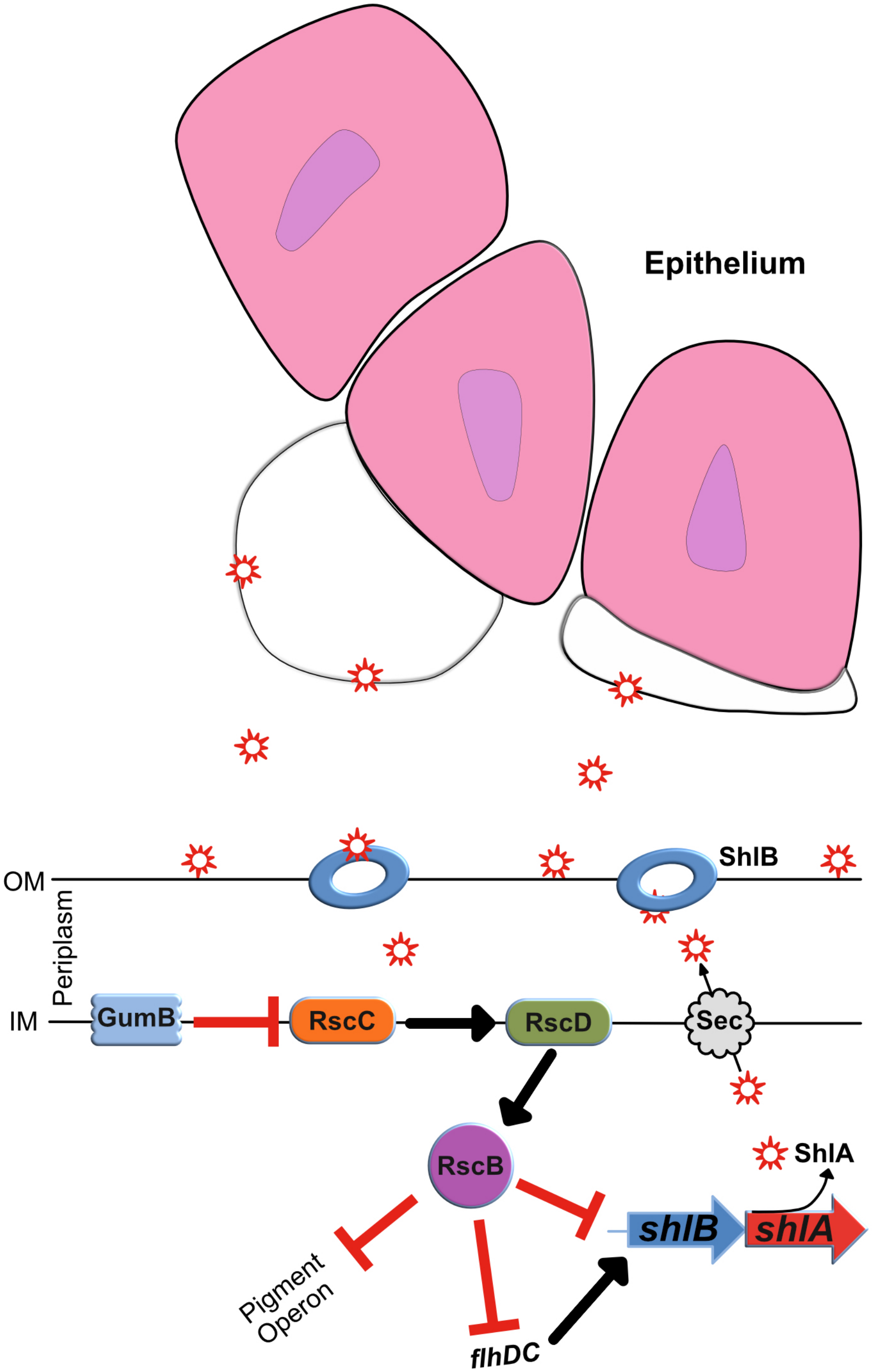

S6 Fig. Model for regulation of *shlBA*. Genetic model for regulation of *S. marcescens* pigment and cytolysin operons. Red bars indicate negative regulation and black arrows indicate activation. Our model predicts that in response to envelope stress, GumB acts as part of the Rcs signal transduction system to modify activity of the RcsB response regulator. In addition to directly inhibiting *shlBA* expression, RcsB also inhibits expression of the *flhDC* operon, which codes for a positive transcriptional regulator of *shlBA*. Expression of the *shlBA* operon leads to secretion of ShlA. Surface associated and surface-released ShlA forms pores in mammalian cells leading to blebbing and finally necroptosis-associated cell death.
