# Supplementary material for: Blowing epithelial cell bubbles with GumB: ShlA-family pore-forming toxins induce blebbing and rapid cellular death in corneal epithelial cells
Source: PLoS Pathog. 2019 Jun 20;15(6):e1007825. doi: 10.1371/journal.ppat.1007825 (PMC6586354; doi:10.1371/journal.ppat.1007825)
Supplement: S7 Fig — The graph represents data from two experiments with cell counts from n≥6 fields of view (n>80 cells per treatment group). HCLE cells treated with GWX 806742X were challenged with wild-type S. marcescens strain K904 at MOI = 50 and after 2 h cells were imaged and bleb frequency was measured. Mean and SD are shown. ANOVA with Tukey's post-test was used and significance is shown by asterisks. * p<0.05, ** p<0.01, **** p<0.0001. Data suggests specific inhibition of necroptosis mediator MLKL reduces bleb formation. (PDF) [file ppat.1007825.s007.pdf]

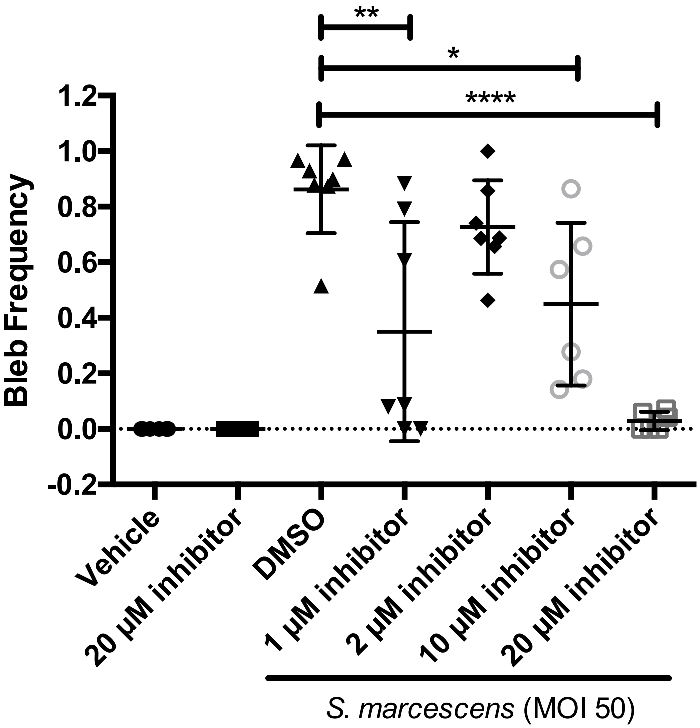

S7 Figure. Inhibition of bleb formation by necroptosis inhibitor GWX806742X. The graph represents data from two experiments with cell counts from n≥6 fields of view (n>80 cells per treatment group). HCLE cells treated with GWX 806742X were challenged with wild-type *S. marcescens* strain K904 at MOI=50 and after 2 h cells were imaged and bleb frequency was measured. Mean and SD are shown. ANOVA with Tukey's post-test was used and significance is shown by asterisks. * $p<0.05$, ** $p<0.01$, **** $p<0.0001$. Data suggests specific inhibition of necroptosis mediator MLKL reduces bleb formation.
