# Supplementary material for: Blowing epithelial cell bubbles with GumB: ShlA-family pore-forming toxins induce blebbing and rapid cellular death in corneal epithelial cells
Source: PLoS Pathog. 2019 Jun 20;15(6):e1007825. doi: 10.1371/journal.ppat.1007825 (PMC6586354; doi:10.1371/journal.ppat.1007825)
Supplement: S8 Fig — Uptake and proliferation of S. marcescens K904 wild type and ΔgumB mutant strains with the vector (pMQ132 and shlBA expression plasmid pMQ541) in RAW macrophage cells (n = 4), mean and standard deviations are shown. Asterisks indicate significant difference by 2-way ANOVA with Tukey’s post-test (* = p<0.05, **** = p<0.0001). (PDF) [file ppat.1007825.s008.pdf]

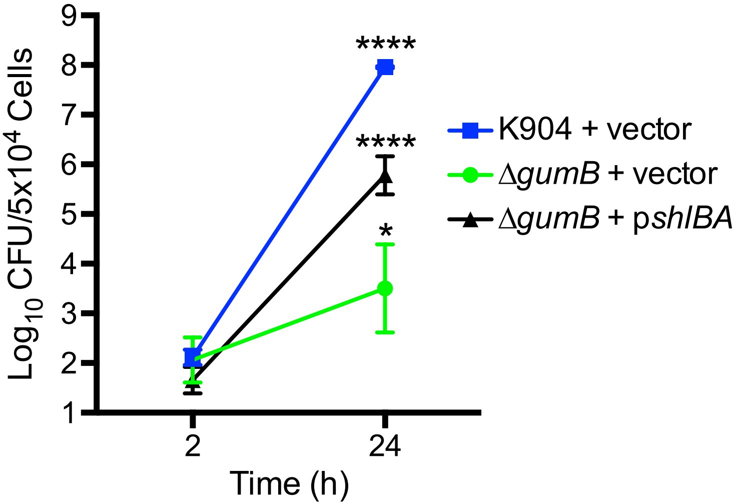

S8 Fig. Role of ShlA in the Δ*gumB* RAW cell proliferation phenotype. Uptake and proliferation of *S. marcescens* K904 wild type and Δ*gumB* mutant strains with the vector (pMQ132 and *shlBA* expression plasmid pMQ541) in RAW macrophage cells (n=4), mean and standard deviations are shown. Asterisks indicate significant difference by 2-way ANOVA with Tukey's post-test (* = $p<0.05$, **** = $p<0.0001$).
