# Supplementary material for: Blowing epithelial cell bubbles with GumB: ShlA-family pore-forming toxins induce blebbing and rapid cellular death in corneal epithelial cells
Source: PLoS Pathog. 2019 Jun 20;15(6):e1007825. doi: 10.1371/journal.ppat.1007825 (PMC6586354; doi:10.1371/journal.ppat.1007825)
Supplement: S1 Table — (PDF) [file ppat.1007825.s012.pdf]

Table S1. Plasmids used in this study

Name	Description	Source or Reference
pBT20	Transposon delivery plasmid	[1]
pSC189	Plasposon delivery plasmid	[2]
pStvZ3	*lacZ* promoter probe plasmid	[3]
pMQ118	suicide-vector *nptII* marker	[4]
pMQ125	*shuttle vector with* P_{BAD}	[4]
pMQ131	*ori*pBBR1, *ori*T, *aphA-3*, *CEN6*/*ARSH4*, *URA3*	[4]
pMQ132	*ori*pBBR1, *ori*T, *aacC1*, *CEN6*/*ARSH4*, *URA3*	[4]
pMQ377	pMQ125 + I-SceI gene	[4]
pMQ460	allelic replacement vector with I-SceI site and *sacB* [5]	
pMQ473	pMQ460 + Δ*shlB* allele	This study
pMQ480	pMQ132 + *gumB*	[6]
pMQ492	pMQ125 + P_{BAD}-*shlBA*	This study
pMQ517	pMQ131 + *gumB*	[6]
pMQ524	*phoA* mariner plasposon delivery plasmid	This study
pMQ529	pMQ132 + *kumO* from *K. pneumoniae*	[6]
pMQ530	pMQ132 + *igaA* from *S. enterica*	[6]
pMQ531	pMQ132 + *yrfF* from *E. coli*	[6]
pMQ541	pMQ492 with P_{nptII}-*shlBA*	This study
pMQ544	*lacZ* promoter probe plasmid with MCS	This study
pMQ553	pMQ544 + *rcsB* internal fragment	This study
pMQ590	pMQ492 with Tn-*phoA* in *shlB*	This study
pMQ591	pMQ492 with Tn-*phoA* in *shlA*	This study
pMQ596	pMQ118 + *hpmA* internal fragment	This study
pMQ600	pMQ132 + *umoB* from P. mirabilis	This study
pMQ601	pMQ125 + *hpmBA* from P. mirabilis	This study
pMQ614	pMQ132 + *rcsB* from *S. marcescens*	This study

pMQ615	pMQ132 + *rcsC* from *S. marcescens*	This study

References:

1. Kulasekara HD, Ventre I, Kulasekara BR, Lazdunski A, Filloux A, Lory S. A novel two-component system contols the expression of *Pseudomonas aeruginosa* fimbrial cup genes. Mol Microbiol. 2005;55:368-80.

2. Chiang SL, Rubin EJ. Construction of a mariner-based transposon for epitope-tagging and genomic targeting. Gene. 2002;296(1-2):179-85. Epub 2002/10/18. doi: S0378111902008569 [pii]. PubMed PMID: 12383515.

3. Kalivoda EJ, Stella NA, Aston MA, Fender JE, Thompson PP, Kowalski RP, et al. Cyclic AMP negatively regulates prodigiosin production by *Serratia marcescens*. Res Microbiol. 2010;161(2):158-67. Epub 2010/01/05. doi: S0923-2508(09)00256-3 [pii] 10.1016/j.resmic.2009.12.004. PubMed PMID: 20045458; PubMed Central PMCID: PMC2846241.

4. Shanks RM, Kadouri DE, MacEachran DP, O'Toole GA. New yeast recombineering tools for bacteria. Plasmid. 2009;62(2):88-97. Epub 2009/05/30. doi: 10.1016/j.plasmid.2009.05.002. PubMed PMID: 19477196; PubMed Central PMCID: PMC2737453.

5. Shanks RM, Stella NA, Hunt KM, Brothers KM, Zhang L, Thibodeau PH. Identification of SlpB, a cytotoxic protease from *Serratia marcescens*. Infect Immun. 2015;83(7):2907-16. Epub 2015/05/06. doi: IAI.03096-14 [pii]10.1128/IAI.03096-14. PubMed PMID: 25939509.

6. Stella NA, Brothers KM, Callaghan JD, Passerini AM, Sigindere C, Hill PJ, et al. An IgaA/UmoB-family protein from *Serratia marcescens* regulates motility, capsular polysaccharide, and secondary metabolite production. Appl Environ Microbiol. 2018. doi: 10.1128/AEM.02575-17. PubMed PMID: 29305504.
