# Supplementary material for: Blowing epithelial cell bubbles with GumB: ShlA-family pore-forming toxins induce blebbing and rapid cellular death in corneal epithelial cells
Source: PLoS Pathog. 2019 Jun 20;15(6):e1007825. doi: 10.1371/journal.ppat.1007825 (PMC6586354; doi:10.1371/journal.ppat.1007825)
Supplement: S2 Table — (PDF) [file ppat.1007825.s013.pdf]

Table S2. DNA oligonucleotide primers used in this study

Primer number	Sequence[a]
2619	ggccagtgccaagcttgcatgcctgcaggtcgactctaCCAAAGCCATATCGCCTCGGTC
2620	ccagtacctgaaccggatcctcGGTGATTTTTTTTATCATTGTAATCCATTATG
2621	cataatggattacaatgataaaaaaaatcaccGAGGATCCGGTTCAGGTACTGG
2622	gataacaatttcacacaggaaacagctatgaccatgaCCTGCAGCAGGCCGTTTTCCACC
2638	AACTGGAGGAAGGTGGGGAT
2639	AGGAGGTGATCCAACCGCA
2664	acgacgttgtaaaacgacgggatctatcatcgtggatccTACTAGTCCGCGGGTCGACCG
2665	gtttcccgactggaaagcgggcagtgagcgcggtcgacccgcggactagtaGGATCCACG
3464	cgaattgggtaccgggccccccctcgaggtcgacggtattcaGGATGCGAAAGACGACGA
3465	ctctctactgtttctccatacccgtaggaggaaaaaATGATAAAAAAAATCACCGCATTG
3639	taagtccccggtctctagaattcaccatagactacaaaCCTGTTCTGGAAAACCGGGCTG
3640	tgtaatcgatatcatgatctttataatcaccgtcatggtcTTATTTCAGCCCCAGAGCGG
3643	gccgctctggggctgaaataaGACCATGACGGTGATTATAAAGATCATGATATCG
3644	attagagcttcaatttaattatatcagttattacccCGACGTTGTAAAACGACGGCCAG
3647	caattcactggccgtcgttttacaacgtcgGGGTAATAACTGATATAATTAAATTGAAGC
3648	cctgctctgcgaggctggccggctaccgccggcgCTTCCCTTCCTTTCTCGCCACGTTCG
3651	ggcgaacgtggcgagaaaggaagggaagCGCCGGCGGTAGCCGGCCAGCCTCGCAGAG
3652	aatatcgccctgagcagcccggttttccagaacaggTTTGTAGTCTATGGTGAATTCTAG
3688	acggccagtgccaagcttgcatgcctgcaggtcgactcTTAGTCTTTGTCCAACGGCGTC
3689	acggccagtgccaagcttgcatgcctgcaggtcgactcTTAGTCTTTGTCCAACGGCGTC
3691	acggccagtgccaagcttgcatgcctgcaggtcgactcTTAGTCTTTGTCCAACGGCGTC
3692	attgtgagcggataacaatttcacacaggaaacagctTTGAAATACTTAGCCTCTTTTCG
3698	cagtgtcaatgcggtgattttttttatcatttttccTCCTCATCCTGTCTCTTGATCAG
3699	atgcagctgggctcatgtttgacagcttaatcgcccgggACGCTGCCGCAAGCACTCAGG
3735	acgacgttgtaaaacgacgggatctatcatcgtggatccTTCACTCTCTTTCGGCGACAG

3736	ggtctagagcggtttcccgactggaaagcgggcagtgaGCTGATCACCGATCTGTCGATG
3892	ttgtaaaacgacggccagtgccaagcttgcatgcctgTTACTGAGGAGGGCGATAAAAAC
3893	ttgtgagcggataacaatttcacacaggaaacagctATGAGATTATCAGTCATTATATTG
3896	ttgtaaaacgacggccagtgccaagcttgcatgcctGGTACGCAAGTGATTAATATAGTC
3897	attgtgagcggataacaatttcacacaggaaacagctatCGTATTAATAATGCGGATACG
3900	aaacgacggccagtgccaagcttgcatgcctgcaggtcgCTATTTTTCAGAAATAGATGC
3901	agcggataacaatttcacacaggaaacagctATGAAATCAAAAAACTTTAAACTTTCACC
3919	aattgggtaccgggccccccctcgaggtcgacggtatcgCTATTTTTCAGAAATAGATGC
3920	tctactgtttctccatacccgtaggaggaaaaaaATGAAAAAAAAAGTTGTTTTATTAAC
4150	GACCTTCAGCGGTTCTCAAT
4151	CTTGGTTTGCTCGGTGTTTG

[a] Upper-case letters indicate sequences that prime amplification of the desired DNA; lower-case letters target homologous recombination with a plasmid. Sequences are listed 5’ to 3’.
